# Real-Time Monitoring of Thermally Induced Twisting–Untwisting of Noncubic Domains in Au Microcrystallites using X‑ray Diffraction Microscopy

**DOI:** 10.1021/acsnano.4c18495

**Published:** 2025-05-20

**Authors:** Chaitali Sow, Abhisakh Sarma, Andreas Schropp, Thomas F. Keller, Dmitry Dzhigaev, Christian G. Schroer, Milan K. Sanyal, Giridhar U. Kulkarni

**Affiliations:** † Chemistry and Physics of Materials Unit, Jawaharlal Nehru Centre for Advanced Scientific Research (JNCASR), Bengaluru 560064, India; ‡ Centre for X-ray and Nano Science CXNS, Deutsches Elektronen-Synchrotron DESY, Notkestraße 85, 22607 Hamburg, Germany; § European X-Ray Free-Electron Laser, 22869 Schenefeld, Germany; ∥ Helmholtz Imaging, 28332 Deutsches Elektronen-Synchrotron DESY, Notkestraße 85, 22607 Hamburg, Germany; ⊥ Department Physik, Universität Hamburg, Luruper Chaussee 149, 22761 Hamburg, Germany; # Deutsches Elektronen-Synchrotron DESY, Notkestraße 85, 22607 Hamburg, Germany; ∇ Saha Institute of Nuclear Physics, Kolkata 700064, India

**Keywords:** scanning X-ray diffraction microscopy, crystal structure, gold, phase transformation, metastable, nanocrystal, twist

## Abstract

Au bipyramids hosting body-centered orthorhombic and tetragonal lattices (bc­(o,t)) exhibit extraordinary stability at ambient conditions and even under high-temperature/high-pressure conditions. The phases undergo conversion to a conventional face-centered cubic (fcc) lattice only during annealing at 700 °C due to the unlocking of the geometrically induced stresses. The spatial distribution of the phases in the crystallite volume has revealed fcc capped bc­(o,t) lattices with two halves of the bipyramid twisted by ∼6° along the length with approximately ± 5% strain. Understanding the spatial distribution and dynamics of these phases at high temperatures can provide detailed information on their thermal stability. Herein, using nanoprobe scanning X-ray diffraction microscopy (SXDM), *in situ* annealing of the bc­(o,t) Au bipyramid (∼1.5 μm long and 300 nm wide) has been performed at different temperatures (up to 800 °C). The study reveals untwisting of the domains assisted by the supplied high temperature, while the existing lattices undergo variation in parameters with negligible changes in proportion. The study reveals and picturizes the dynamic change in diffracting volumes across a wide temperature range. Notably, despite annealing, ∼83% of the bc­(o,t) content is still retained (with different lattice parameters), proposing the annealing route to produce unusual metastable lattices of gold.

## Graphical abstract

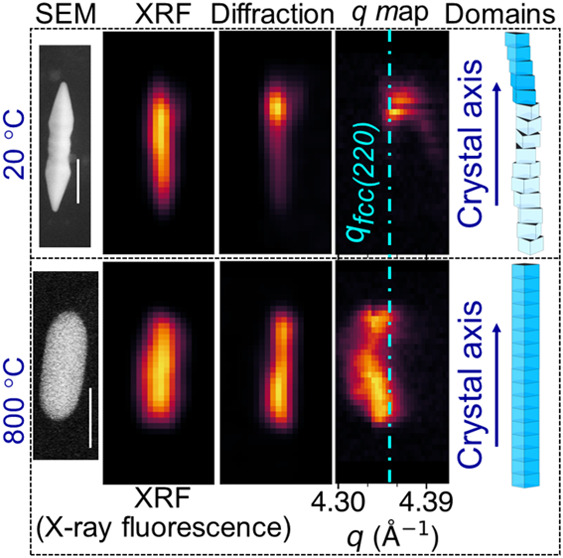

Bulk gold (Au) crystallizes in an fcc lattice and demonstrates extraordinary oxidant-resistant behavior,^1^ remarkable optical and electrical properties,^2^ and a high work function.^3,4^ Therefore, stabilization of Au in unconventional lattice structures has been a subject of matter for a few decades. There have been a few reports on the phase transformation of bulk Au to hexagonal closed-packed (hcp) and body-centered cubic (bcc) lattice structures, under high pressure^5^ and high shock compression,^6,7^ respectively. However, at ambient conditions, the non-fcc lattice structures in bulk Au are not yet known. So far, few reports have led the path by stabilizing Au in non-fcc lattices at the nanoscale, *e*.*g*., the hcp (2H and 4H) phase of the Au square sheets,^8^ nanoribbons,^9^ nanokites,^10^ nanorods,^11,12^ nanowires,^13–16^ and nanostars.^17^ Besides, during the growth of Ge nanowires, the Au catalyst at the tip of the nanowire is found to crystallize in the 2H lattice.^18^ Another report demonstrates the growth of hcp Au nanostructures on a single-crystal Ge(001) surface.^19^ Besides, e-beam and CO gas-assisted phase transformation of fcc to 4H in the epitaxially grown fcc Au nanoparticles on the 4H nanorod template has also been shown.^20^ Au nanocrystals in body-centered tetragonal (bct) phase have been reported under mechanical deformation.^21^ Our previous work demonstrated stabilization of Au microcrystallites in the form of body-centered orthorhombic and tetragonal lattices (together, bc­(o,t)),^22^ and these crystallites act as an excellent catalyst.^23^

Understanding the stability of the metastable lattices under harsh chemical and physical perturbants is crucial for real applications. For example, surface ligand exchange induces lattice transformation in 2H Au square sheets.^24^ In the presence of adsorbates/oxidizing agents, the bc­(o,t) crystallites undergo phase transformation to fcc even at relatively lower temperatures.^25–27^ Among the many known physical perturbants responsible for causing phase transformation, the widely used ones are temperature, pressure, and e-/ion-beam irradiation.^28,29^ The temperature and pressure-led phase transformation to fcc has been observed in 4H Au nanoribbons,^30^ nanowires,^13^ and e-beam induced transition, in the case of 2H Au square sheets.^8^ The bc­(o,t) Au crystallites, which are stable under ambient conditions,^22^ are also stable under e-beam application (200 keV).^23^ Additionally, low-energy Ar^+^ ion exposure on the bc­(o,t) crystallites results in other unusual body-centered tetragonal (bct*-*I) phases.^29^ A previous report shows thermal stability of bc­(o,t) Au at ∼400 °C, while a phase transformation to fcc at elevated temperature (∼700 °C) was observed by *ex situ* scanning electron microscopy (SEM) and laboratory X-ray diffraction (XRD) (on the microscale).^31^ During the phase transformation to fcc, the crystallites undergo deformation in the morphology due to the self-diffusion of Au atoms. Besides, the crystallites display a reversible phase transformation under an applied pressure ∼40 GPa.

Herein, we study the Au microcrystallites with ∼92% bc­(o,t) phases.^27^ Stabilization of the bc­(o,t) phases in the microcrystallites is mainly favored by the geometrical constraints induced by the penta-twinned bipyramidal morphology.^22^ Under proper growth kinetics, the unlocking of stresses is manifested in the stabilization of the bc­(o,t) lattices. Theoretical calculation reveals that the strains are within the elastic regime (∼1%).^22^ Under high-temperature treatment, the metastable phases convert back to stable fcc, which is linked to the relaxation of strains. The strain relaxation is faster at higher temperatures.^31^ Due to the presence of bc­(o,t) lattices, these microcrystallites act as catalysts for the *p*-nitrophenol reduction reaction^23^ while remaining inactive in Hg.^32^ Furthermore, the existence of these unconventional lattices in the crystallites results in nonhomogenous Cu deposition when subjected to a Cu electroless plating medium.^32,33^

Thus, monitoring the phase transformation *via in situ* annealing with high spatial resolution should yield interesting observations. Further, understanding the intermediate states during annealing is important for the stabilization of different lattice parameters within the same crystallite, which may find applications in catalysis. The bc­(o,t) Au crystallites studied here are ∼300 nm thick and, therefore, not suitable for transmission electron microscopy (TEM)-based studies. Besides, the focused ion-beam (FIB)-based sectioning method is destructive for crystallites.^34^ Because of the high penetration depth of X-rays, nanometer-sized synchrotron hard X-ray beams allow the nondestructive visualization of thick objects (∼a few μms) in great detail. A current scanning X-ray diffraction microscopy (SXDM) study with a 100 nm beam of a bc­(o,t) Au crystallite has revealed the presence of large strain (simultaneously, −6.06% and +4.21%) and strain anisotropy among the domains.^34^ Additionally, the crystallite body is twisted (∼6°) along length. These phases are kinetically arrested in the crystallite volume, and thus, annealing at high temperature tends to release the stresses by stabilizing the thermodynamically favorable fcc lattice.^22^ Therefore, strain engineering of the same crystallite would be possible with annealing as the controlling parameter. Earlier efforts to study the effects of thermal annealing on a collection of crystallites have revealed the generation of unusual body-centered tetragonal lattices, eventually causing lattice transformation to fcc.^31^ As the study was based on a laboratory setup, the information made available was limited. Herein, we report *in situ* annealing measurements of a non-cubic Au bipyramid at high temperatures while collecting SXDM diffraction data from the same crystallite. In order to study the *in situ* strain relaxation at the nanoscale at high temperatures (up to ∼800 °C) while keeping the sophisticated experimental setup unaltered (such as a small working distance of a few mm), an MEMS-based resistive heater was used, where the heating at the metallic coil remained local (no significant heating beyond ∼35 μm, see Figure S1). From the *in situ* diffraction data, many interesting observations, such as annealing induced untwisting of the domains, and variation in the lattice parameters of the non-cubic lattices, have prevailed. Additionally, an *in situ* study at 800 °C for a prolonged duration showcases the presence of a large anisotropic strain across the crystallite body.

## Results and Discussion

The as-synthesized bc­(o,t) crystallites were dispersed in acetone and drop-casted on a Si/SiN_*x*_ membrane-based nanochip heater. The middle of the heater hosts a thin SiN_*x*_ membrane (500 × 500 μm^2^) suitable for the passage of the X-ray beam, as shown in Figures S1 and S2a–c. Prior to the diffraction experiment, a fast raster scan was performed with 15 keV X-rays (0.827 Å wavelength and 100 nm circular beam) in 50 × 50 nm^2^ steps to find the Pt-based localization markers in the vicinity of the preselected Au crystallite by monitoring Pt L_α_ and L_β_ X-ray fluorescence (XRF) lines at 9.4 and 11.07 keV, respectively (see Figure S3). Later, the crystallite placed vertically near the marker was located using Au L_α_ and L_β_ lines at 9.7 and 11.4 keV, respectively. The chosen crystallite was nearly parallel to the *z*-axis of the goniometer, as shown in Figure 1a–c. The selected crystallite, ∼1.5 μm in length, was raster-scanned with the beam rather slowly (for 1 s/scan point) in a scan area of (1 × 2) μm^2^ across the *y*–*z* plane, as shown in Figure S4. The morphology of the crystallite appeared in the fluorescence map, as shown in Figures 1d and S5.

**Figure 1:**
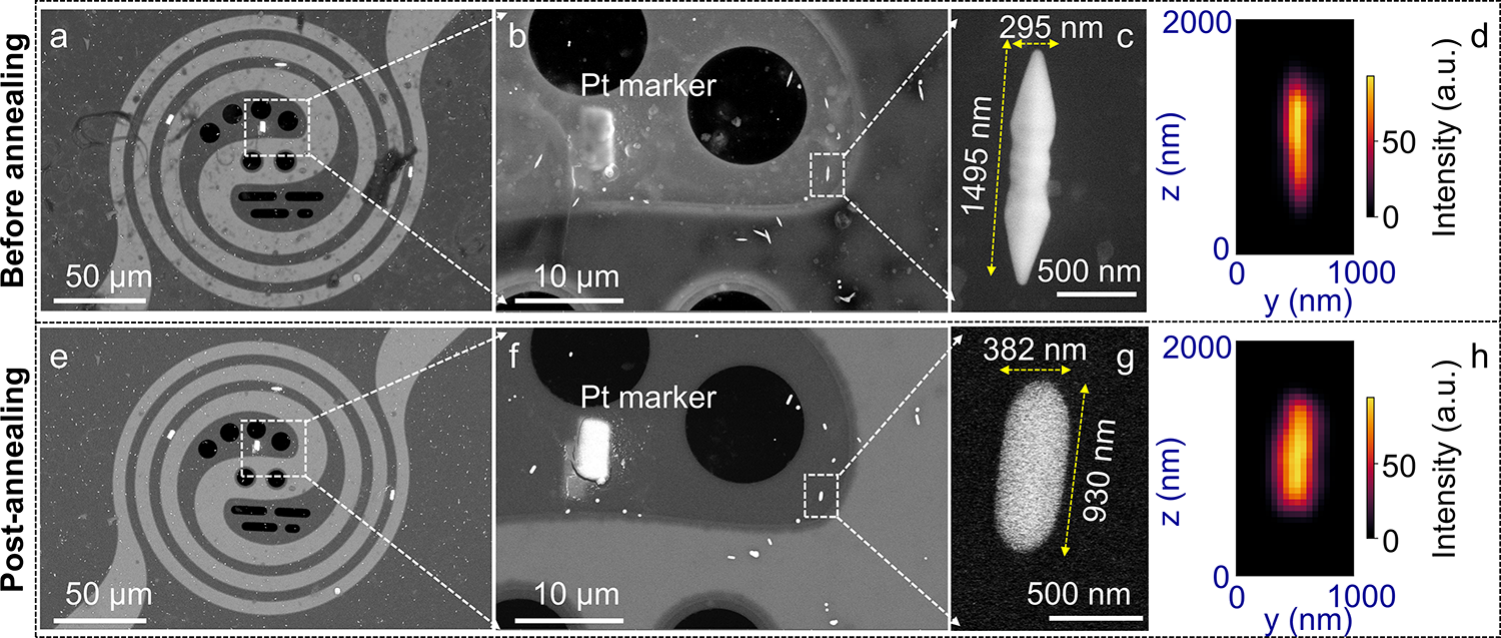
Monitoring the Au microcrystallites on the SiN_*x*_ membrane before and after annealing. (a) SEM image of the predeposited Pt markers (white rectangles, ∼5 × 10 μm^2^) on the SiN_*x*_ membrane along with Au microcrystallites. (b, c) Magnified SEM images of the studied region of the membrane and crystallite. The chosen crystallite was nearly parallel to the Pt marker and thus nearly vertically aligned in the experimental setup. (d) X-ray fluorescence (XRF) map of the crystallite based on Au L_α_ and L_β_ lines shows the crystallite morphology. (e–g) SEM images of the heater, marker, and crystallite after annealing at 800 °C. The images are clearer as residual organic contaminants from the precursor are removed during annealing. The Pt marker is distinctly visible, whereas the morphology of the crystallite changes during the process (compare c and g). The XRF map post-annealing (h) and that before annealing (d) also follow this trend.

The crystallite morphology and its position on the grid were monitored during the entire experiment using the XRF map, verifying the presence of crystallite within the defined scanning window (Figure S5). The temperature of the heater coil was elevated by supplying a current (following the calibration data supplied by the manufacturer; see the device in Figure S6). The required temperature of the heater (such as 180 °C) was achieved within ∼2 min of supplying a constant current. After attaining the desired temperature, the current was supplied continuously for 10 min, which resulted in continuous heating at 180 °C for 10 min. Then, the current was gradually reduced (in ∼5 min) until the temperature reached 20 °C (see Figure 2a). The SXDM data was recorded from the crystallites at 20 °C. Similarly, other temperatures (300, 400, 500, 600, 700, and 800 °C) were achieved by following the above-mentioned process by varying the current while retaining other experimental conditions similar. On a few occasions, the mesh scan was repeated to obtain a better view/resolution. This also helped us to understand the effect of the time gap on annealing. Toward the end, the crystallite was heated to 800 °C and held at this temperature so as to collect one data set at an elevated temperature. The measurement routine, such as annealing temperature (°C), annealing time (min), and data collection (at 20 or 800 °C and time-lapse data collection), is defined in Figure 2a, and the same notation has been used throughout the manuscript.

**Figure 2:**
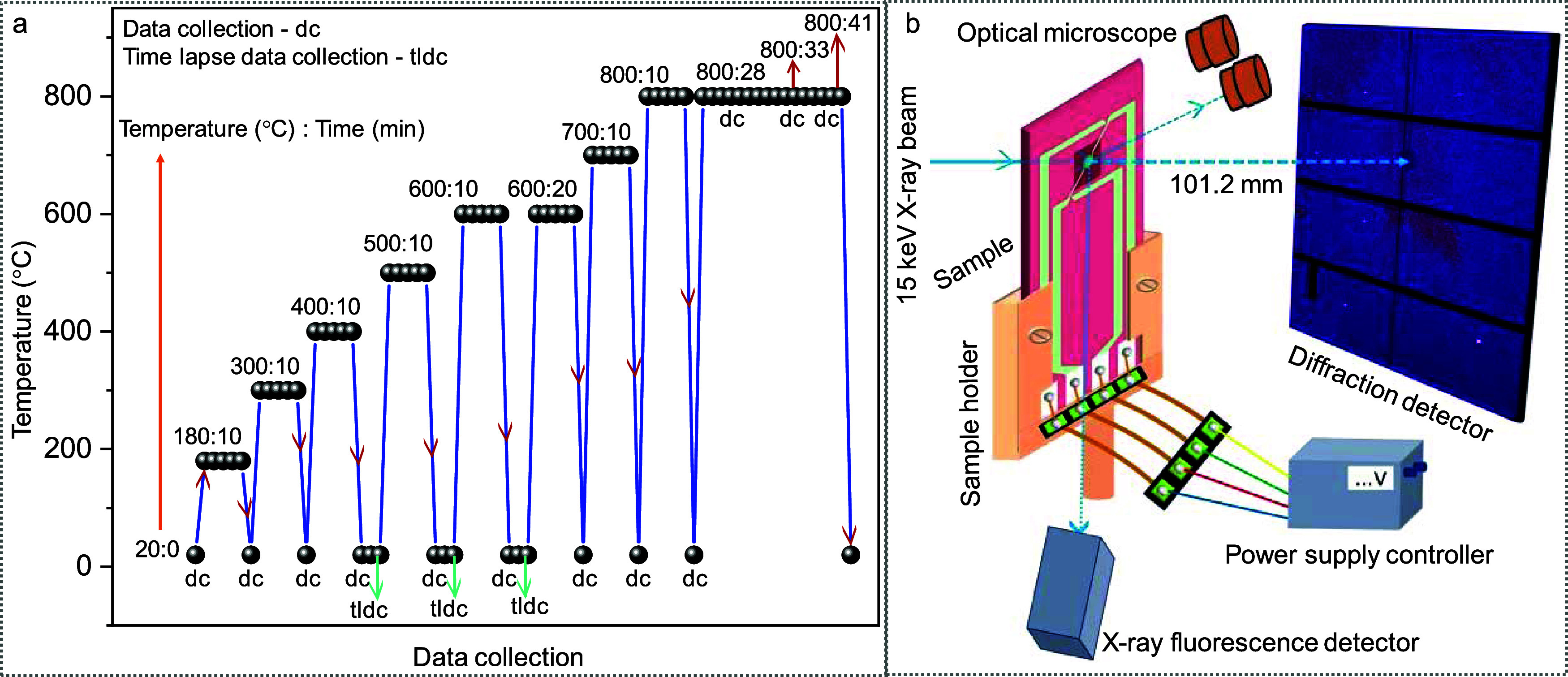
Data collection schemes. (a) Annealing and data collection routines. (b) Schematic diagram of the experimental setup for scanning X-ray diffraction microscopy (SXDM). In most of the cases, data was collected (dc) at 20 °C, although the last three data sets were collected at 800 °C while annealing continued at that temperature. Besides, in the cases of 400, 500, and 600 °C (with 10 min annealing), data were collected two times at 20 °C. The latter data collection point is denoted as time-lapse data collection (tldc). The annealing temperature and treatment time details are represented as temperature (°C):treatment time (min).

Annealing-induced deformation in the morphology of the nanostructures is quite common.^13,31^ The Pt marker appeared brighter due to annealing (see SEM images in Figure 1e,f), while the studied Au crystallite exhibited a rounded, thicker shape as though the corrugations corresponding to high-index facets as well as tips were smoothened (Figure 1g,h). Compared to the preannealed crystallite in Figure 1c (1495 nm in length and 295 nm in width), the crystallite is now ∼930 nm in length and 382 nm in width, *i.e*., a reduction in the area by ∼9%, as observed from the projection in the image (see Figure 1g). The surrounding crystallites also underwent similar changes in morphology (Figure 1b,f), thereby confirming that crystal deformation is induced by heating. This rounding of the crystallite has been observed previously *via ex situ* heating at 700 °C.^31^ Such morphological changes can be expected primarily at less coordinated sites on high-index facets and sharp tips due to the self-diffusion of the Au atoms aided by an ample supply of thermal energy (see Figure S7).^31^ Crystallites lying far away from the heater region maintained the bipyramidal morphology (Figure S8).

The diffraction data was recorded over the mesh scan using an Eiger X 4M detector located at a distance of 101.2 mm from the crystal center (Figures 2b and S9). The pristine crystallite was rotated along the crystal axis until a few spots concurrently satisfied the diffraction condition and appeared in the detector. We confirmed by SXDM measurements that for each reflection, the diffracting volume moves over the crystallite when rotated due to the presence of twisted domains, as found earlier.^34^ We discuss the evolution of the twisted domains as a function of annealing. Therefore, appropriate conditions (mesh scan parameters, crystal position, and angle) were locked and used throughout the experiment. Concurrently, the diffraction and fluorescence data sets were recorded with a collection time of 1 s per scan point (see Figures S3, S5, and S10). At 20 °C, the diffraction data recorded from the pristine crystallite showed six bright spots in different detector segments (Figures S10 and 3a). Data collection and analysis strategies are shown in Figure 3b–d. During the analysis, the diffraction spots and, therefore, the line profiles were integrated (*i.e*., summed up the collected diffraction patterns) along the *y* (Figure 3c) and along both the *y* and *z* directions (Figure 3d).

**Figure 3:**
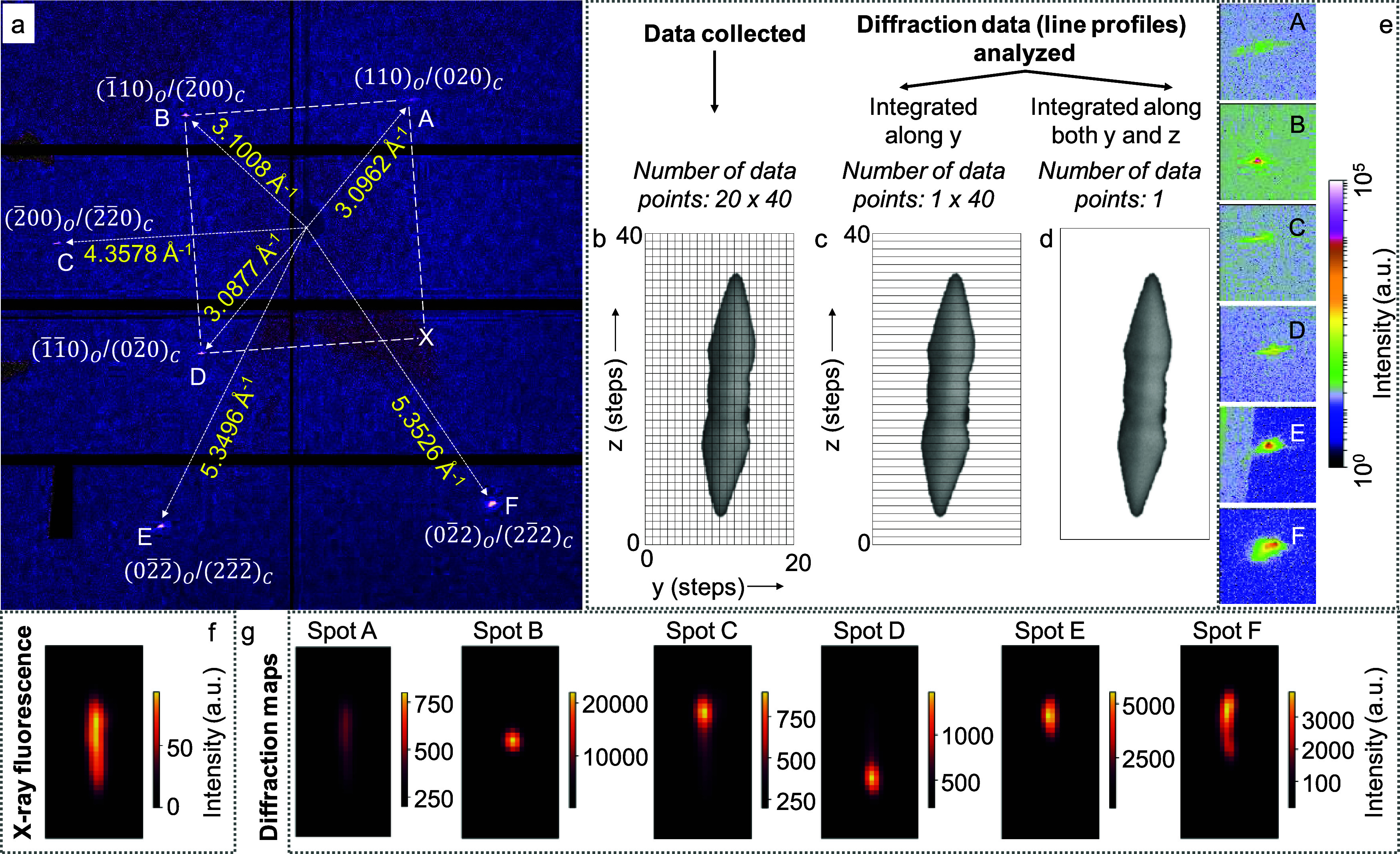
(a) Integrated diffraction data obtained from the pristine crystallite using an Eiger X 4M detector at 20 °C (background subtracted). The spots were indexed considering the orientation of the unit cells, as shown in Figure S11. A white rectangle is drawn to connect the symmetry-related {110}_O_/{020}_C_ spots (X denotes the location of the symmetry reflection, if it appeared). C and O refer to face-centered cubic (fcc*)* and body-centered orthorhombic (bco*)* lattices, respectively. (b-d) Data collection and analysis schemes. (b) Mesh scan of the Au crystallite with a scan step of 50 × 50 nm^2^. Integrated scan points along (c) the *y*-direction and (d) both the *y* and *z* directions. (e) Magnified view of various diffraction spots in (a), with an area size of 100 × 100 pixels. (f) XRF map of the crystallite. (g) Diffraction maps of various diffraction spots, highlighting the parts of the crystallite that satisfy the diffraction condition.

The integrated (both y and z) diffraction spots (obtained by summing all the diffraction patterns collected on the detector, Figure 3d) and their line profiles are shown in Figure 3e and Table S1, respectively. The integrated intensities were dispersed (see details in Table S1), and the centroids of the line profiles were used for the determination of *q* values (Table S1, within the allowed detector resolution of ± 0.0045 Å^–1^). The *q* values for reflections A, B, and D are 3.0962, 3.1008, and 3.0877 Å^–1^, respectively. The spots are nonuniform, and the quantified spreads along the radial and azimuthal directions are large (Figure 3e and see Table S2). Here, for the sake of simplicity, we consider only fcc and bco lattices. Using the CrystalMaker software package, the diffraction spots were simulated by rotating both the bco and fcc unit cells along the crystal axis by 360° with the *b-*axis of bco coinciding with the rotation axis, while the *c-*axes of fcc and bco were parallel to each other (see details in Figure S11a). The generated patterns were then overlaid (Figure S11b). Here, the simulation was restricted to a *q* radius of 2.66 to 4.37 Å^–1^. Considering the *q* spread for reflections A, B, and D, these values are comparable to those exhibited by bco{110} and fcc{002} reflections (see details in SI, Tables S3 and S4). The three spots appear to form three corners of a near-rectangle, as shown in Figure 3a, with the angles between the diagonals being 83.2–89.85° and 90.35–95.2°, respectively. Therefore, the spots seem to appear from a family of reflections and can be assigned to {110}_O_/{002}_C_. The manifestation of symmetry reflections, despite no crystal rotation, is striking and will be discussed in later paragraphs.

Spot C appears in the horizontal line on the detector plane (see Figure 3a) and possesses a *q* value of 4.3578 Å^–1^ with a large radial spread (∼0.1238 Å^–1^). Therefore, spot C can be assigned to {200}_O_/{220}_C_ (see details in Table S4). The *q* values for reflections, E and F, are ∼5.3496 and 5.3526 Å^–1^, respectively, which are assigned to {022}_O_/{222}_C_. To gain insight into the characteristics of the diffraction spots, circles possessing *q* of fcc were drawn (Figure S12). The arcs of the circles are shown in white and pass through the diffraction spots. Interestingly, widespread spots are clearly visible with large distributions over the arcs, implying a large strain in the pristine crystallite. Hence, each spot is designated *q*_min_ and *q*_max_, covering the full area of the spot. From the observed spread, the derived lattice parameters of bco are *a*_min_ = 2.9090 Å, *b*_min_ = 3.0011 Å, and *c*_min_ = 3.8483 Å (calculated using *q*_min_ values); *a*_max_ = 2.8280 Å, *b*_max_ = 2.7885 Å, and *c*_max_ = 4.2327 Å (calculated using *q*_max_ values); see Tables 1 and S5. Therefore, the variations in the bco lattice parameters are Δ*a* = 0.0810 Å, Δ*b* = 0.2126 Å, and Δ*c* = −0.3844 Å. The changes in volume/two atoms in the bco lattices with respect to fcc are −1.1393% and −1.7805%.

**Table 1:** Calculated bco Lattice Parameters at 20 and 800 °C^a^

temperature (°C)	bco	*a*_bco_ (Å)	b_bco_ (Å)	*c*_bco_ (Å)	volume (Å^3^)	change in volume/two atoms (with respect to fcc −33.9836 Å^3^) (%)	*c*/*a*	*c*/*b*
20	bco_min_	2.9090	3.0011	3.8483	33.5964	–1.1393	1.3228	1.2822
	bco_max_	2.8280	2.7885	4.2327	33.3785	–1.7805	1.4967	1.5179
800	bco_min_	2.9588	3.0089	3.8587	34.3529	1.0867	1.3041	1.2824
	bco_max_	2.8326	2.7800	4.2838	33.7333	–0.7365	1.5123	1.5409

a The number of atoms in bct generated from the fcc unit cell (i.e., c/a = 1.414) is 2 (see Figure 6b for details). Similarly, the number of atoms in bco is 2. In order to compare the volume of different unit cells, the volume per two atoms is considered.

In order to correlate the simultaneous presence of symmetry reflections (such as spots A, B, and D), spot C and other symmetry reflections (spots E and F), the XRF and diffraction maps are shown in Figure 3f,g. The geometrical locations of the diffracting volumes (at 20 °C) are different; for example, the top tip contributes essentially to spot A, the central part to spot B, and toward the bottom tip to spot D. Similarly, toward the top tip, the diffraction conditions for spots C, E, and F are satisfied. Careful consideration of the nature of the diffraction spots/maps suggests that the sources of symmetry reflections can be different and even unique (see Figure 3g). The crystallite hosts penta-twinned geometry, and therefore, the simultaneous satisfaction of symmetry reflections without any further crystal rotation is linked to the differently oriented domains of the 5-fold symmetric crystal (for details, see Figure S13a). Spots E and F appear simultaneously from another part of the crystal, again due to the penta-twinned geometry (Figure S13b). The appearance of {110}_O_/{002}_C_ (spots A, B, and D), {200}_O_/{220}_C_ (spot C), and {022}_O_/{222}_C_ (spots E and F) symmetry reflections enable to visualize the fcc and bco unit cell orientations within the crystallite volume. For example, the bco *b-*axis coincides with the crystal axis, the *c-*axes are parallel to each other, and the fcc unit cell is rotated with respect to bco by 45°, as shown in Figure S11a (also see Figure 3 in ref 34). Therefore, the unit cell orientation demonstrates the growth directions as bco <010> and fcc <110> (see Figure S11a) with a large strain within.

The diffraction data recorded from the crystallite annealed at 180 °C exhibits nearly unaltered spread in the diffraction spots and their corresponding diffracting volumes (see Figures 4 and S14). Upon further increasing the temperature to 300 °C, the diffraction data introduced significant changes in the spots (see Figures 4 and S14). Specifically, for spot D, the spreads are ∼0.1441 Å^–1^ (radial) and 2.85° (circumferential). These effects are even visible in the diffraction maps (see Figure 4c) as enhancements in the diffraction volumes of the spots. Such enhancements are general observations and not specific to a particular {hkl}, as shown in Figures 4c and S14g. Upon further increasing the temperature to 400 °C, the spots exhibit larger spreads (see Figure S15), for example, in spot C (radial ∼0.1388 Å^–1^ and circumferential ∼0.55°), spot D (0.1116 Å^–1^, 2.3°), and spot E (0.0890 Å^–1^, 1.65°). In general, a large radial spread signifies the contribution of the variation in lattice parameters, while a large circumferential spread refers to differently oriented domains or unit cells. Here, the large spread in Figures 4b,d,f and S14 can be explained by comparing them with the powder pattern (Figure S16) collected from a collection of annealed crystallites (at 400 °C), which is also in line with our earlier report on thermal annealing of collection of crystallites.^31^ The pattern collected from the annealed crystallites (*ex situ*) exhibits broad (002) reflection, as shown in Figure S16 (at 2θ ∼44.5°).^31^ During annealing at 400 °C, the incorporation of an unusual body-centered tetragonal phase (bct-I) has been seen with different lattice parameters than the pristine bct.^31^ Similar observations can be invoked here with the *in situ* annealing effect (see Figure 4).

**Figure 4:**
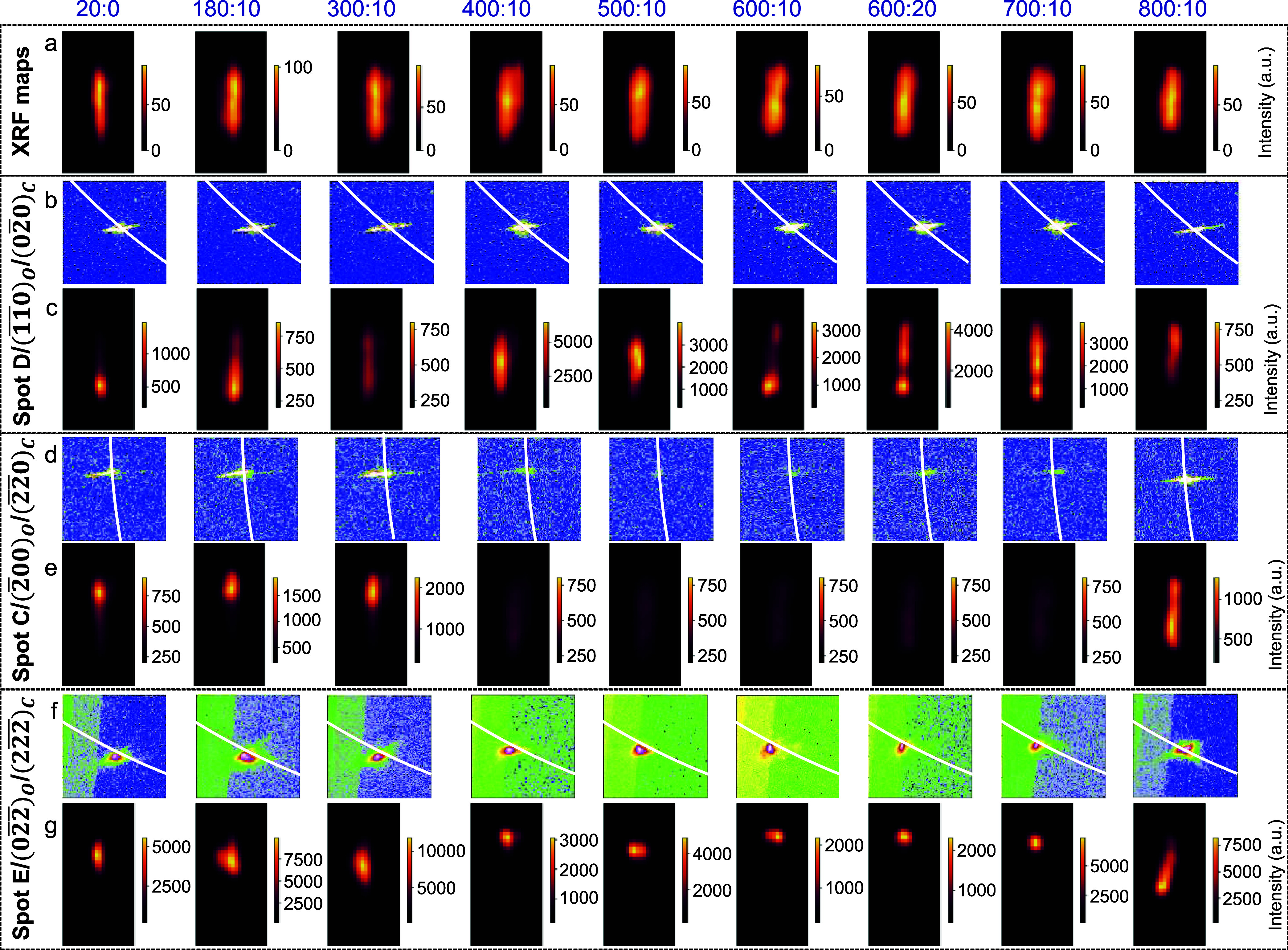
Temperature-dependent (a) XRF maps, (b, d, f) diffraction spots D, C, and E, and (c, e, g) the corresponding diffraction maps. Pixel windows (100 × 100) are shown in (b, d, f). The temperature and time details are shown on top. The white arcs of the circles, which correspond to the *q* values of fcc(002), fcc(220), and fcc(222), pass through the spots. The *q* values were derived from the standard fcc Au lattice parameters (3.0804, 4.3564, and 5.3355 Å^–1^; see details in Table S3).

Further, the temperature was elevated in steps of 100 °C from 400 to 800 °C. The corresponding collected diffraction data represent large radial spreads, hinting at the presence of large strains with respect to fcc (see Figures 4, S14, and S15). With increasing annealing temperature, the circumferential spreads also become significantly higher, which, along with the radial spread (*i.e*., circumferential spread 2.5 to 3.2° and radial spread 0.1302 to 0.1677 Å^–1^ for spot D), refers to the presence of multiple lattices. The increase in the radial spread signifies the distribution of lattice parameters, which is intriguing compared with a conventional bc­(o,t) to fcc phase transformation with an expectation of a significant reduction in volume contribution. The XRF maps of the crystallites exhibit deformation in the morphology, a dumbbell-like feature that can be correlated with the SEM image in Figure 1g (collected post-annealing experiment).

An observation drawn from the diffraction maps is that the diffracting volume satisfying Bragg’s condition increases irrespective of the spot. Specifically, the enhancement is noticeable starting from 300 °C (Figures 4c,e,g and S14c,e,g). Therefore, the emerging diffraction volume can be linked to the reduction in twist around the crystal axis, possibly along the entire length of the crystallite (see Figure 5). This is in agreement with the annealing-induced enhancement in crystallinity, which arises due to the growth of larger grains and deformation in the morphology.^31^ With annealing, a reduction in the inherent twist occurs, importantly, in a coherent manner such that the grains mend in the same orientation (Figures 4e and 5b,d). The untwisted domains concurrently favor diffraction conditions, leading to an increased diffraction volume (Figure 5b). This can be visualized clearly from the *ex situ* temperature-dependent SEM images of the crystallite, shown in Figure S7. The reduction in corrugation is prominent with an increase in temperature and nearly disappears at 700 °C.

**Figure 5:**
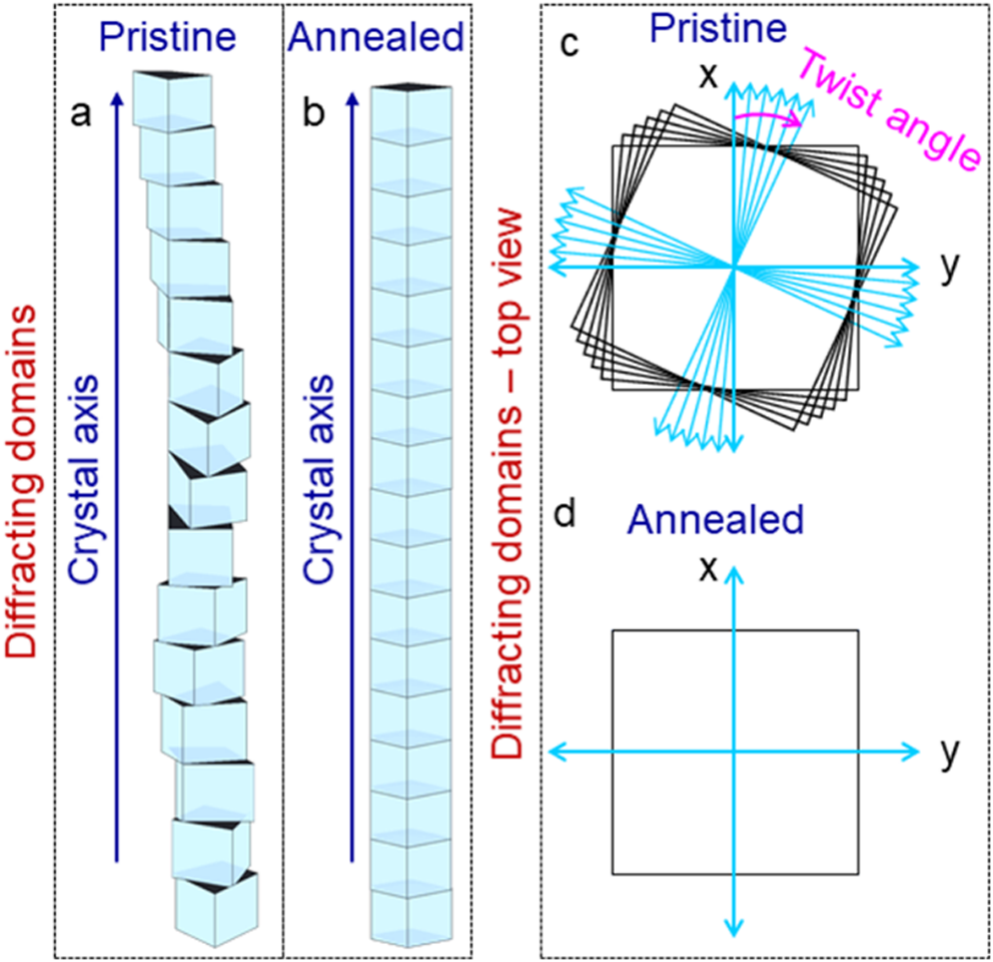
Schematic representation of (a, b) twisted and untwisted crystallite domains across the crystallite length, respectively. (c, d) Top views of the twisted and untwisted domains, respectively. The presence of twists within the crystallite domains hinders the satisfying of Bragg’s diffraction condition for the full length. Annealing-induced untying of the twist along the same direction with respect to the crystal axis enhances the diffraction volume. Therefore, satisfying the diffraction condition of the full crystallite length (in annealed crystallite) would require minimal crystal rotation along its axis.

To understand the changes in the spot intensity pattern, line profiles are drawn along the radial direction for the spots, integrated along both the *y* and *z* directions, as shown in Figures 6a and S17. Spot D appears in all of the recorded data and also while continuously heating at 800 °C (following the scheme in Figure 2a). The variation in integrated line profiles of spot D at different annealing temperatures is shown in Figure 6a. The profiles exhibit visible changes starting from 300 °C, and with a further increase in temperature, the change becomes prominent with increased line broadening or in the form of multiple peaks. The line profiles show a significant change in the peak position (see Figure 6a); the pristine crystallite hosts negative Δ*q *= *q*_fcc_ –* q*_bco_, while the annealed crystallite (after 800 °C – 800:10) accommodates positive Δ*q* values. Thus, a change in the *q* trend from negative to positive is evident. A similar peak shift was noticed in spots B-E (Figure S17b–d). From the *q* values, the bco lattice parameters were calculated (Tables 1 and S5). The changes in volume/two atoms in the bco lattices at 800 °C are 1.0867% and −0.7365% (with respect to fcc), which is remarkably different from pristine crystallite (*i.e*., −1.1393% and −1.7805%). Therefore, the overall reduction in volume at 800 °C suggests a phase transformation, although it is not complete. Further, the *c*/*a* and *c*/*b* ratios are different compared to the as-prepared crystallite (see Table 1). The variation in *c*/*a* and *c*/*b* ratios is the key for the generation of bco from fcc (*c*/*a* or *c*/*b* = 1.414 for fcc), as suggested by the Bain transformative path^35^ and also shown in Figure 6b,c; therefore, reverting back to fcc would be expected to follow a similar trend.

**Figure 6:**
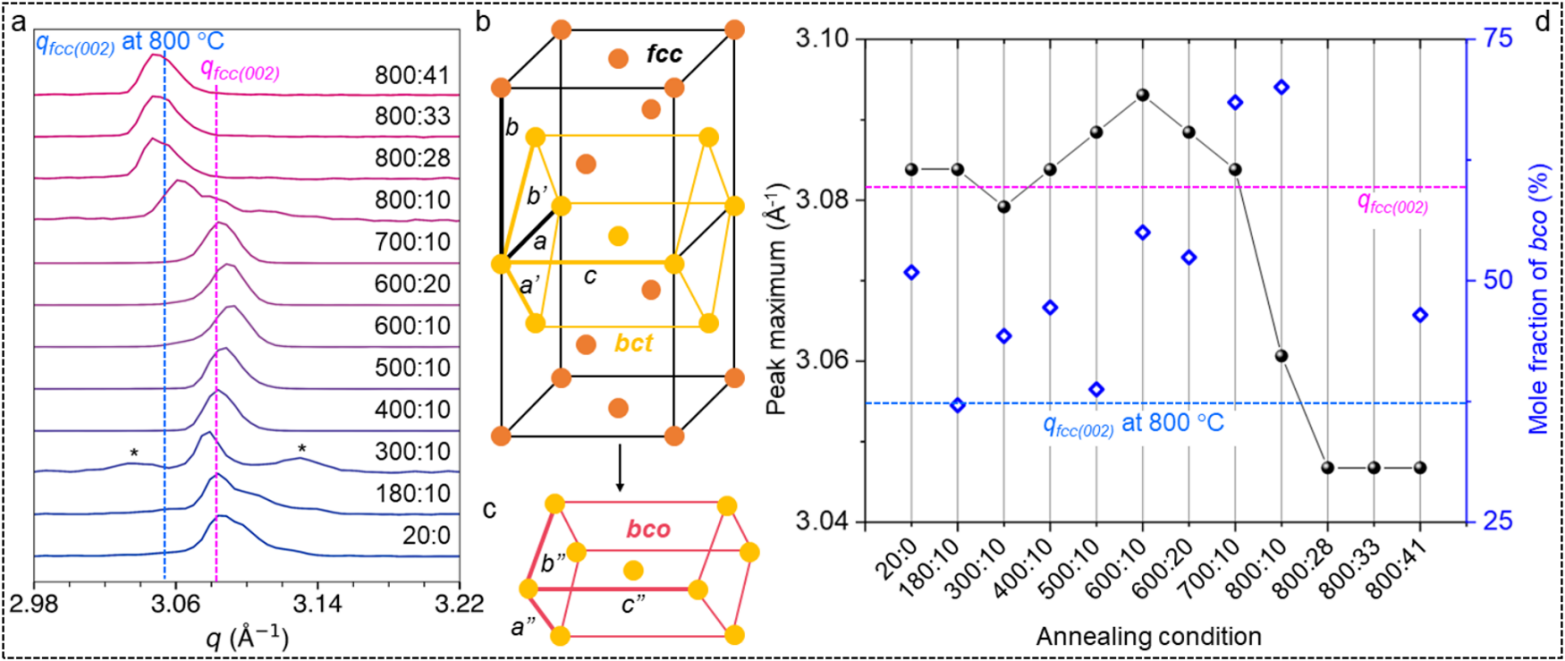
(a) Integrated (along both *y* and *z*) line profiles (normalized) drawn over spot D at different annealing temperatures. Clearly, the peak position shifts with the annealing conditions. At first, *q* shifts to a higher side and then a large shift to the lower side at 800 °C (800:10). The patterns collected while heating at 800 °C (800:28, 800:33, and 800:41) show a further shift toward lower *q*. To understand this, the thermal expansion of Au at 800 °C was considered (blue dashed line). * denotes an additional emerged peak. (b, c) Schematic representation of the generation of bco from the fcc lattice. bct refers to the body-centered tetragonal lattice with $a^{\prime }=\frac{c}{\sqrt{2}}$ . Variations in *a’*, *b’*, and *c* parameters result in the bco lattice. (d) Peak position (maximum intensity) and bco mole fraction plotted at different annealing conditions. These values were extracted from the integrated diffraction data in (a). The pink (*q* of fcc at 20 °C) and blue (*q* of fcc at 800 °C) dashed lines are drawn for reference.

In order to understand the effect of heating, concurrent heating and recording of data at 800 °C for 28, 33, and 41 min were done (see Figure 2a). Interestingly, the peak position of spot D at 800 °C (800:28, 800:33, and 800:41) shows a further positive shift (Figure 6a). To understand this extensive large shift, the thermal expansion of Au at 800 °C (*i.e*., 0.76%)^36^ was taken into consideration (*i.e*., fcc(002) = 3.0572 Å^–1^). Although the latter does not solely contribute toward it (see the blue dashed line), the additional positive Δ*q* is due to the presence of different lattices at 800 °C, which is noticeably different from conventional fcc. The presence of this large positive shift in *q* at 800 °C (after 41 min) demonstrates the robustness of the existing metastable lattices at high temperatures. Hence, the peak positions of the maximum intensity of the line profiles are plotted in Figure 6d. Interestingly, the noticeable change in the *q* trend starts at 600 °C (3.0884 Å^–1^) and reaches a minimum at 800 °C (3.0605 Å^–1^), *i.e*., 0.75% lower with respect to 20 °C (3.0838 Å^–1^). Additionally, the variation in the mole fraction of bco at different annealing temperatures is also plotted in Figure 6d. The bco proportion was quantified from the area under the curve of the line profiles (see details in Figure S18). Surprisingly, the overall proportion of bco (51%) remains similar to that at 600 °C (55% and 52%, respectively); however, it increases at 700 °C to 68% and further to 70% at 800 °C. The concurrent occurrence of the retention of the bco proportion while the alteration in the peak position signifies a change in the lattice parameters without any modification in the overall bco proportion. Further, the increase in the bco proportion directly relates to the enhancement of the increased diffracting volume enriched with bco lattices. Therefore, treatment at 800 °C allows stabilization of bco lattices with much different lattice parameters while retaining a similar bco proportion. The bco mole fractions calculated from spot C and spot E at 800 °C are 83% and 55%, respectively (Figure S19). The annealing-induced enhancement of the diffracting volume is maximum in the case of spot C, arising from the plane parallel to the crystal length (*i.e*., growth direction) and, therefore, the quantified maximum bco content after 800 °C is considered as 83%. Further, concurrent heating and data collection at 800 °C for 41 min results in 46% bco (from spot D). Although the full crystallite length satisfies diffraction (Figure S20), the reduction in the bco proportion signifies a phase transformation to fcc. Hence, annealing-induced phase transformation is neither a complete transformation nor a direct conversion. However, the continuous formation of different phases and their gradual changes in the intensity distributions are the observations reflected in the *in situ* annealing (see the line profiles of the spots in Figure S17). It should be noted here that the bco proportion was quantified from a single incident angle theta; therefore, the extracted proportion based on the integrated peak intensities obtained by theta scanning can be different.

Annealing-induced enhancement of the diffraction volume prompts us to use the experimental details of spatially resolved nano-diffraction, and spot C is well suited for this demonstration. Hence, the diffraction spots are integrated only along the *y*-direction (as shown in Figure 3c), resulting in spots at different locations (*z*) of the crystal. The line profiles drawn over the integrated spot C (along *y*) at each location *(i.e*., by summing all of the diffraction patterns collected on the detector at the same *z*; Figure 3c), the two-dimensional (2D) map of *q* vs *z* (different steps along the crystal length) was plotted across the total scanning window in Figure 7. The XRF map, diffracting volume (from the diffraction map), and its *q* value (2D map) can be compared (see Figure 7a–c). For example, in the as-prepared crystal, the top tip of the crystal (spot C, with *q* ∼4.3611 Å^–1^) hosts fcc*-*rich lattices (see Figure 7b,c). Due to the presence of twist, only a small part of the crystal (at 20 °C data) satisfies Bragg’s condition (Figure 7d). In contrast, at 800 °C, nearly the full crystal length satisfies Bragg’s condition (Figure 7e–g), showcasing the disappearance of twists (Figure 7h). Besides, post-annealing at 800 °C introduces non-uniformity in the *q* spread (see Figure 7g) along the length. Specifically, it showcases that the center (∼4.3311 Å^–1^) is strained compared to the tips (∼4.3467 Å^–1^) by 0.35%, although the overall crystallite is untwisted (see Figure 7g). This observation supports a strained body with less-strained tips and demonstrates the co-presence of compression and expansion (Figure 7g). Therefore, information on the presence of metastable lattices even after annealing at 800 °C prevails in spot C. This demonstrates the superior capabilities of the nanoprobe SXDM technique in revealing the strain distribution and dynamics in the studied thick single Au crystallites compared with other routinely used techniques such as TEM and full-field X-ray diffraction microscopy.

**Figure 7:**
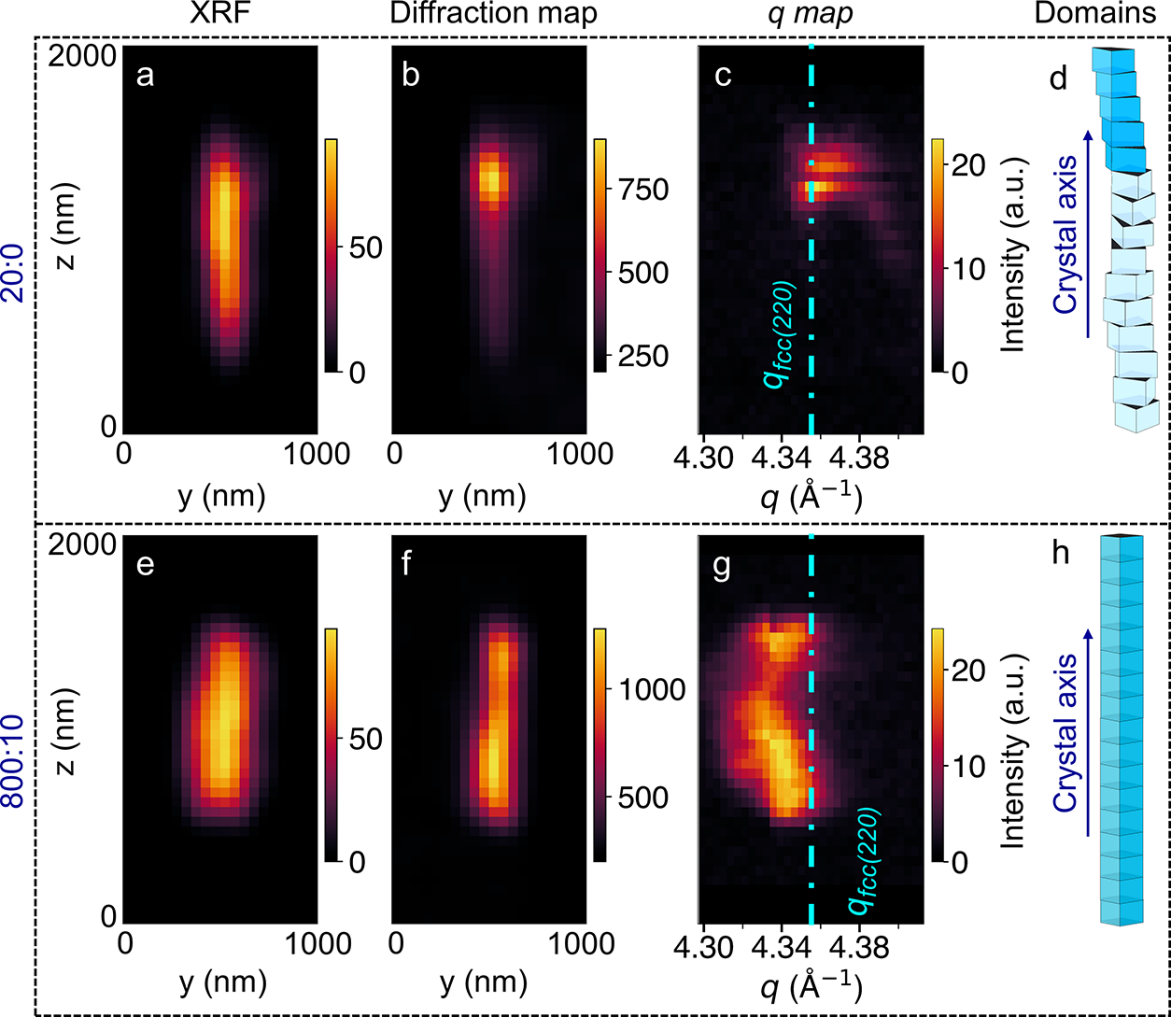
(a, e) XRF maps of the studied crystallite and (b, f) diffraction maps of the spot *C*/(2̅00)_*O*_/(2̅2̅0)_*C*_ at different annealing conditions. (c, g) 2D maps of *q* *vs* *z* for spot C at different annealing conditions. *q* *vs* intensity obtained from the integrated line profiles (along *y*) for all *z* steps (as shown in Figure 3c) were used to create the 2D map. (d, h) Schematic representations of twisted and untwisted domains (as already shown in Figure 5a,b) within the crystallite volume. The domains contributing to diffraction are in dark blue color. Clearly, the entire length of the annealed crystallite satisfies the diffraction condition. The Turquoise dash-dotted line represents *q* of fcc. The annealing conditions are shown on the left side of the panels.

The diffraction data at temperatures of −400, 500, and 600 °C were collected immediately after cooling and after some time lapse (see Figure 2a), where the latter is referred to as well settled at ambient conditions (Figure S21). Both of these diffraction maps (immediately collected and time-lapse collected) do not match, and surprisingly, the time-lapse data shows a reduction in the intensity of diffraction spots and their corresponding diffraction maps compared to those obtained immediately after cooling (Figure S21c for 400 °C). The enhancement/reduction in the diffraction volume is directly related to the presence of twists within the crystallite; therefore, the shrinkage of the diffraction volume in the cooled/well-settled crystallites hints at the possibility of reoccurrence or reverting back of the twists toward the original situation (Figure S21). The scenario repeats at 500 °C, where data collected immediately after cooling shows the disappearance of twists; in other words, enhancement of the diffraction map area. However, a similar reduction in the diffraction volume (as was seen in the case of 400 °C) can be witnessed again in the well-settled post-annealed crystallite. Importantly, in the time-lapse images, the untwisting/twisting is prominently seen post 600 °C treatment, and therefore, the untwisting/twisting must be governed by kinetics.

The pristine bc­(o,t) microcrystallites act as catalysts for the *p*-nitrophenol to *p*-aminophenol reduction reaction in contrast to conventional bulk fcc Au.^23^ The catalytic performance monotonically increases with increasing proportion of bc­(o,t). The performance of the catalyst was attributed to the uplifting of the *d*-band center in the bc­(o,t) lattices, and also a reduction in the effective coordination number.^16^ The annealed crystallites host unconventional lattices (even at 800 °C) with a range of different lattices. As a result, the co-presence of these different lattices is expected to promote the catalytic performance of the crystallites. Considering these points, the presence of various metastable lattices in the crystallite volume would be interesting to study for the strain-/crystal-structure-dependent reactivity. Therefore, *in situ* annealing of the crystallite would pave the way for an alternate path for strain engineering of metastable lattices. This study opens up the possibility of tuning the catalytic performance by fine-tuning the strain within the catalyst volume.

## Conclusions

In summary, the SXDM study showcases the presence of large strain in the pristine bc­(o,t) Au bipyramid with bco <010> and fcc <110> as the growth directions. Further, *in situ* studies have addressed the strain relaxation process. The inherent twists among the diffracting domains undergo untwisting during annealing and, interestingly, in the same direction, such that the diffraction volume increases. The untwisting of the domains is reversible at a lower temperature (∼500 °C) and, therefore, dependent on annealing kinetics. At the end of the annealing experiment, the co-presence of large tensile and compressive strains reveals the strain relaxation process toward fcc, through the alteration in *c/a* and *c*/*b* ratios. Additionally, the spatially resolved study allows visualization of the rotation of domains and reduction in twists along the crystal axis, which are otherwise difficult to depict and further difficult to understand. Although the bco lattices are expected to be metastable, the presence of 83% bco after annealing at 800 °C demonstrates the commendable stability of these lattices post-annealing at 800 °C. Post-annealing, the central part of the crystallite is highly strained compared to the tip regions. The annealed crystallite hosts a mixture of Au polymorphs and can, therefore, be used for the growth of different kinds of phases and further examine their structure–property relationships. Besides, annealing can be used as a controlling parameter to stabilize unusual metastable lattices. Finally, this study demonstrates the exceptional capabilities of SXDM for the visualization of strain gradient across the nanocrystallite body, even under *in situ* conditions.

## Materials and Methods

### Synthesis of Au Microcrystallites

Tetraoctylammonium bromide (ToABr), aq ammonia, silver nitrate (AgNO_3_), and hydrogen tetrachloroaurate (III) hydrate (HAuCl_4_.3H_2_O) were obtained from Spectrochem, India, and used as procured. Hydrochloric acid (35%) and toluene were obtained from SD Fine Chemicals, India. The Au microcrystallites were synthesized following a previously reported recipe.^27^ Briefly, 300 μL of 50 mM ToABr, 75 μL of 25 mM HAuCl_4_, and 30 μL of 25 mM AgNO_3_ were added, and the mixture was stirred for 5 min. In this mixture, 100 μL of 35% (v/v) HCl was mixed and further stirred for another 15 min. The phase-transferred Ag­(I) and Au­(III) ions in the toluene medium were isolated and used as precursors. The thermal decomposition of the precursor at 250 °C results in Au microcrystallites. In order to remove the unreacted precursor and Ag contaminants, the prepared microcrystallites were washed with toluene, followed by ammonia.

### Characterization of Au Microcrystallites

*Ex situ* XRD data was collected using a PANalytical instrument (Cu Kα, 1.5406 Å; scan rate 3°/1 h). Similarly, temperature-dependent changes in the crystallite morphology (*ex situ*) were monitored using an Apreo 2 SEM (Thermofisher (FEI)).

### Au Microcrystallites on SiN_*x*_ Nanochips with an In-built Heater

The as-synthesized crystallites were dispersed in acetone and drop-casted onto a single-tilt nanochip membrane (SiN_*x*_) with an in-built heater from Wildfire nanochip (DENS solutions for Thermo Fisher Scientific (FEI) microscopes, P.T.H.SS.1).Table 2

**Table 2:** Specification Details of the Heater

temperature accuracy	>95%
temperature homogeneity (within a central diameter of ∼100 μm)	>99.5%
temperature stability	<0.005 °C
bulging at 750 °C	negligible
bulging at 1300 °C	<5 μm
drift rate	<0.3 nm/min
sample displacement from RT to 1000 °C	<200 nm

### Sample Preparation

The heater consists of 6 circular and 4 rod-shaped holes (Figure 1a), where the SiN_*x*_ membrane is ultrathin. The nearly vertically aligned crystallites placed on the membrane of the nanochip were located using a dual-beam FIB/SEM^37^ (see Figures 1a,b and S3). The crystallites on top of the metallic coil or outside the coil were not considered. The heater along with the differently shaped holes serve as markers, as they are easily discernible in an optical microscope (100× magnification at the beamline). In order to assist further, particularly with locating the small-sized crystallites (∼1.5–3 μm length), Pt markers (4 in number) were deposited of each size, 5 × 10 μm^2^ within the proximity (∼20 μm) of the vertically aligned crystallites (see Figure S2d) using electron-beam-induced deposition (EBID).^37^ Thus, Pt markers produced could be tracked by using XRF maps (Figure S3).

### Fabrication of the Device

(a) Customizing the Substrate Holder: The substrate holder was designed and manually cut in such a way that the nanochip fits well in the holder. At the same time, the four contact pads are reachable for making contacts as well as remaining unhampered during SXDM measurements. At the bottom of the holder, a PCB board is attached for the electrical contacts (see Figure S6). (b) Making Contact: The Au microcrystallite-loaded membrane was used for making contacts. From the four prefurbished contact pads, four thin (∼40 μm) Cu wires were used to establish connections between the four contact pads of the Wildfire nanochip and the PCB board. Ag-epoxy (cured at 120 °C in air for an hour) was used for making contacts using Cu wires with the contact pads of the Wildfire chip and Sn–Pb solder for making contacts with the four pads of the PCB board. Afterward, four thick (∼100 μm) insulated Cu wires were soldered to the four contact pads of the PCB (see Figure S6). According to their use, these four connecting wires were connected to the required instrument module (Keithley 4200–SCS semiconductor Characterization System and DENS-E-03–00 Digi-heater control box). (c) Measurement of Resistance: After completion of two rounds of making contacts from the contact pad to the PCB board and from the PCB board to the power supply controller, the device was studied using a Keithley 4200-SCS Semiconductor Characterization System in the four-probe measurement configuration. The 4-probe measurement shows that the resistance of the heater is ∼200 Ω.

### Interfacing the Heater with the Software for *In Situ* Control

The calibrated heater was connected to a temperature controller procured from DENS Solutions. The controller is pre-programmed to monitor the temperature of the heater while remaining outside the X-ray hutch. Calibrated temperature vs. resistance data (obtained from the manufacturer) were used at the time of the experiment.

### Performing Scanning X-ray Diffraction Microscopy (SXDM)

The SXDM experiment was performed at beamline P06 (nanoprobe endstation) at the PETRA III synchrotron radiation source (DESY, Germany). Nanofocusing lenses (NFLs) out of silicon were used to focus the beam on the sample.^38^ An energy-dispersive X-ray fluorescence detector (VORTEX EM silicon drift detector) was positioned at an angle of 90° with respect to the beam and closer to the sample for locating the desired Au microcrystallite. An Eiger X 4M detector (Dectris Ltd., pixel size: 75 × 75 μm^2^) was positioned at 101.2 mm downstream from the sample to measure the wide-angle X-ray scattering signal (see details in Figure 2b). The beam was characterized by ptychography using a resolution text chart (model ATN/XRESO-50HC, NTT-AT). The studied sample was scanned using a focused 100 nm X-ray beam in 50 nm steps over an area of 1 × 2 μm^2^ (see Figure S4). The X-ray optics unit, along with the laser interferometers (for precise position control of the sample), enabled to monitor the sample position with respect to the optics in the scanning microscopy experiment.^39,40^

### Further Details about the Data Collection Scheme

During data collection, there were times when some scans were repeated in the pursuit of a better view/resolution. This process has given some room to observe the intermediate processes, if any changes have occurred. For example, the crystal is annealed for 10 min at a specific temperature and then immediately cooled down to 20 °C in 5 min. Then, the data were collected (Figure 2a). If any scan was repeated, then the repetition was done after that. This eventually gave some time and provided ample opportunity for the crystal lattices/twists to undo the processes if they were made to undergo changes. Therefore, this allowed us to compare the effects of this time gap.

Scans where data collection was done at elevated temperature were performed in a larger grid with faster scan parameters, such as 40 × 40 steps with 0.1 s/step acquisition time. The scanning time was normalized for all the scans to 1 s/step. Additionally, the grid size was cropped to (20 × 40 steps) to match the rest of the data.

### Data Analysis

The diffraction data were analyzed using custom Python scripts and DPDAK.^41^

### Simulating the Diffraction Pattern

The CrystalMaker software package was used for the generation of simulated diffraction patterns.

## Supplementary Material



## Notes

Nanochip with in-built heater; SEM images of nanochip loaded with Au crystallites and Pt markers; SEM and XRF images of the Pt marker; mesh scan measurements; XRF map of crystallites at different annealing conditions; image of nanochip with contacts in it; *ex situ* temperature-dependent SEM images of a crystallite; SEM images of the heating coil and the crystallites inside the coil at different magnifications; schematic representation of the diffraction geometry; integrated diffraction data collected at different annealing conditions; visualization of the unit cells and simulating the corresponding reflections; diffraction spots along with fcc circles with *q* of (002), (220), and (222) passing through the spot; SEM image of penta-twinned tip of the crystal along with twin boundaries; temperature-dependent XRF maps; diffraction spots A, B, and F and the corresponding diffraction maps; calculation of the spread of the various diffraction spots at different annealing conditions; laboratory XRD patterns of bc­(o,t) Au crystallites annealed at 400 °C along with the pristine pattern; line profiles of the diffraction spots (integrated along both y and z) A, B, C, E, and F, respectively, drawn along the radial direction at various temperatures; peak fitting of spot D at different annealing conditions; peak fitting of spots C and E after 800 °C; concurrent heating and data collection of spot D at 800 °C−temperature-dependent XRF maps, diffraction spots and the corresponding diffraction maps; time-lapse data collection (tldc) and temperature-dependent XRF maps, diffraction spots and the corresponding diffraction maps of spot D at 400, 500, and 600 °C; estimating *q* values and *q* spreads before annealing; {*hkl*} and *q* values for fcc, bct, and bco lattices, respectively; comparison of the obtained *q* values with different lattices, and calculated lattice parameters of the bco crystallite (PDF)
