# Supplementary material for: Real-Time Monitoring of Thermally Induced Twisting–Untwisting of Noncubic Domains in Au Microcrystallites using X‑ray Diffraction Microscopy
Source: ACS Nano. 2025 May 20;19(21):19635–45. doi: 10.1021/acsnano.4c18495 (PMC12139044; doi:10.1021/acsnano.4c18495)
Supplement: Supplementary file 1 [file nn4c18495_si_001.pdf]

Supporting Information

Real-Time Monitoring of Thermally Induced Twisting–Untwisting of Noncubic Domains in Au Microcrystallites using X-ray Diffraction Microscopy

Chaitali Sow,[1,2*] Abhisakh Sarma,[3] Andreas Schropp,[1,4] Thomas F. Keller,[1,5] Dmitry Dzhigaev,[6] Christian G. Schroer,[1,4,5] Milan K. Sanyal[7] and Giridhar U. Kulkarni[2*]

[1]Centre for X-ray and Nano Science CXNS, Deutsches Elektronen-Synchrotron DESY, Notkestraße 85, 22607 Hamburg, Germany

[2]Chemistry and Physics of Materials Unit, Jawaharlal Nehru Centre for Advanced Scientific Research (JNCASR), Bengaluru 560064, India

[3]European X-Ray Free-Electron Laser, 22869 Schenefeld, Germany

[4]Helmholtz Imaging, Deutsches Elektronen-Synchrotron DESY, Notkestraße 85, 22607 Hamburg, Germany

[5]Department Physik, Universität Hamburg, Luruper Chaussee 149, 22761 Hamburg, Germany

[6]Deutsches Elektronen-Synchrotron DESY, Notkestraße 85, 22607 Hamburg, Germany

[7]Saha Institute of Nuclear Physics, Kolkata 700064, India

Email: sow.chaitali325@gmail.com; chaitali.sow@desy.de; kulkarni@jncasr.ac.in

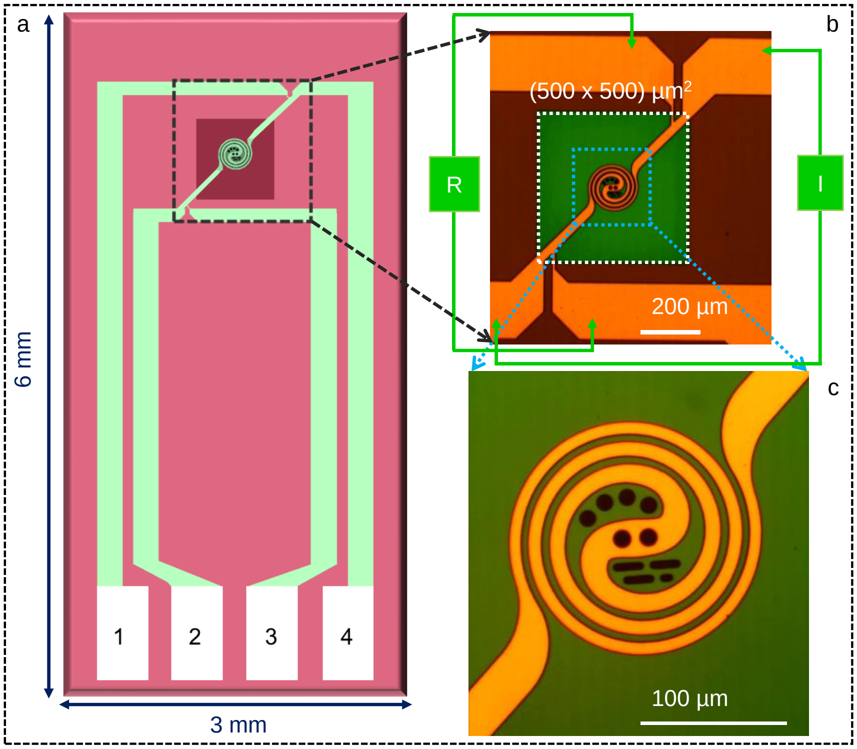

Figure S1. Nanochip with in-built heater. (a) Schematic representation of the as-procured nanochip with an in-built heater. The four contact pads are shown in white. (b) Optical image of the heater along with connections required for the 4-probe measurement. The nanochip consists of a (500 × 500) μm^2 SiN_x membrane area (in green) within which a resistive heater (orange) is present. The contact pads (2,4) and (1,3) (as shown in (a)), used for applying current (I) and measuring resistance (R), respectively during the heating experiment. (c) Optical image of the heater, where the orange and greenish colors are the metallic coil and SiN_x membrane, respectively. At the central region, circular (6) and rod-shaped (4) regions are present where SiN_x membrane is ultrathin.

in (a).

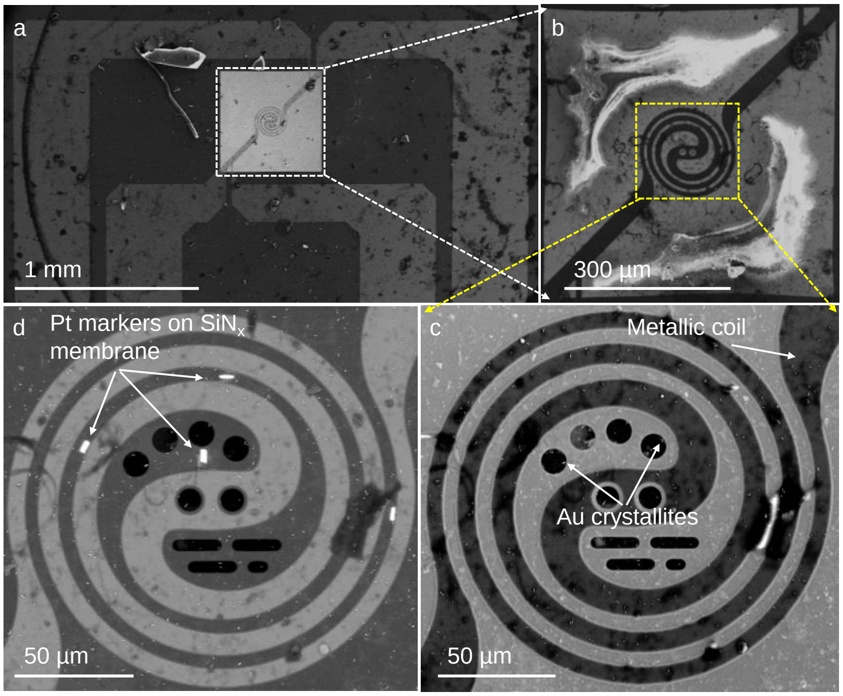

Figure S2. (a-c) SEM images of Au crystallites loaded nanochip at different magnifications displaying the SiN_x membrane, heater and the crystallites. The microcrystallites were drop-casted on the heater region of the membrane as shown in Figure S1. (d) SEM image of the nanochip in (c) with Pt markers in it. The metallic coil and holes are easily locatable by optical microscope (100× magnification at the beamline). Thus, only small sized four Pt markers (~ 5 × 10 µm^2) near the vertically oriented crystallites were fabricated on the SiN_x membrane (in the greenish region in Figure S1c). These markers were located using X-ray fluorescence (XRF) signals of Pt L_α and L_β lines, as detailed below under Figure S3.

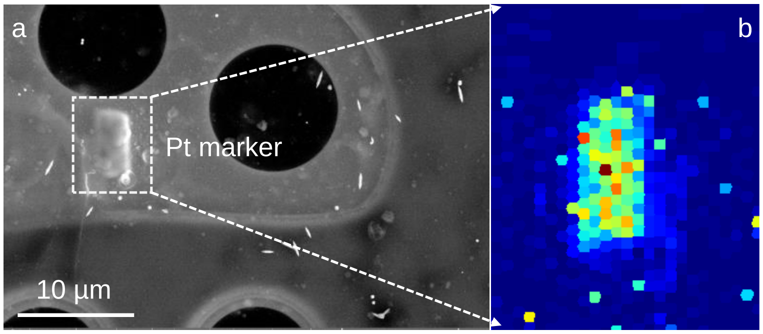

Figure S3. Pt marker. (a) SEM image of the Pt marker on SiN_x nano-chip membrane (5 × 10 µm^2 in size). (b) Integrated XRF image of Pt L_α and L_β lines collected from the white dashed rectangle in (a).

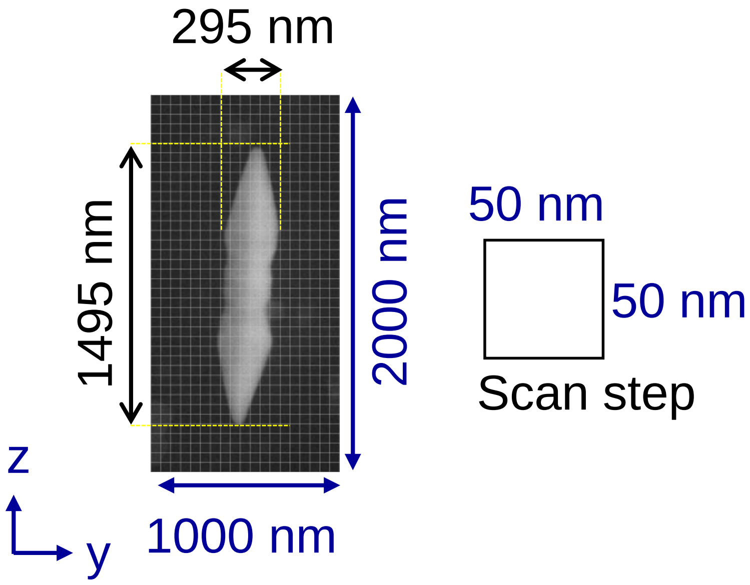

Figure S4. Mesh scan measurement. Data was collected from a 1000 (y) × 2000 (z) nm^2 grid encompassing the chosen crystallite. Step size, 50 × 50 nm^2 (20 rows along y and 40 columns along z). The mesh parameters were optimized based on the beam diameter (100 nm), the crystallite size and the data acquisition time.

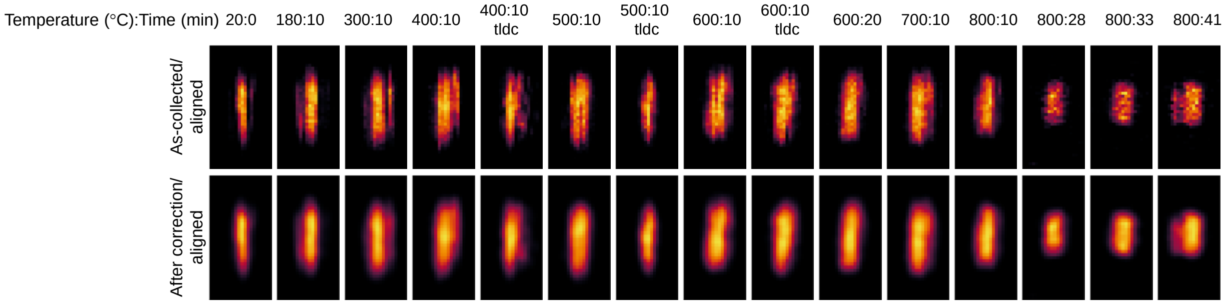

Figure S5. XRF map correction. During the experiment, the sample/beam was shaking occasionally leading to blurring of XRF maps. Therefore, the maps are corrected implementing Gaussian filter. Later, the similar corrections were applied in the diffraction maps, in order to reduce the fluctuation caused by vibration of sample/beam. Note, the morphology of the crystallite changes with increasing annealing temperature hinting the possible deformation during annealing as was previously observed in another study.[1] Annealing and data collection details are written at the top of the panel (also, see Figure 2a for details). tldc refers to time lapse data collection.

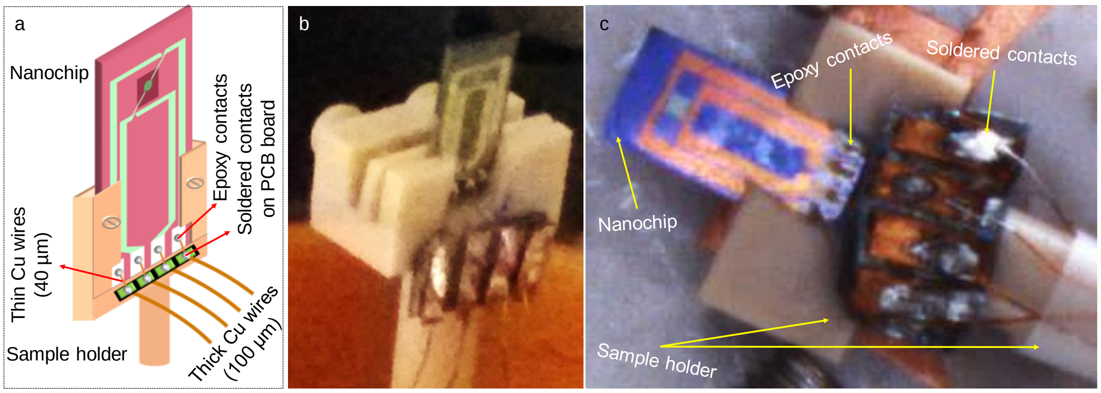

Figure S6. (a) Schematic representation of the nanochip, mounted on the customized sample holder, with contacts to connect with the power supply controller. (b) Photograph of the nanochip. (c) Photograph of the nanochip mounted on the sample holder collected while imaging inside SEM (during the post-annealing data collection process). The temperature could be fine-tuned and accurately set through the computer-controlled calibrated software.

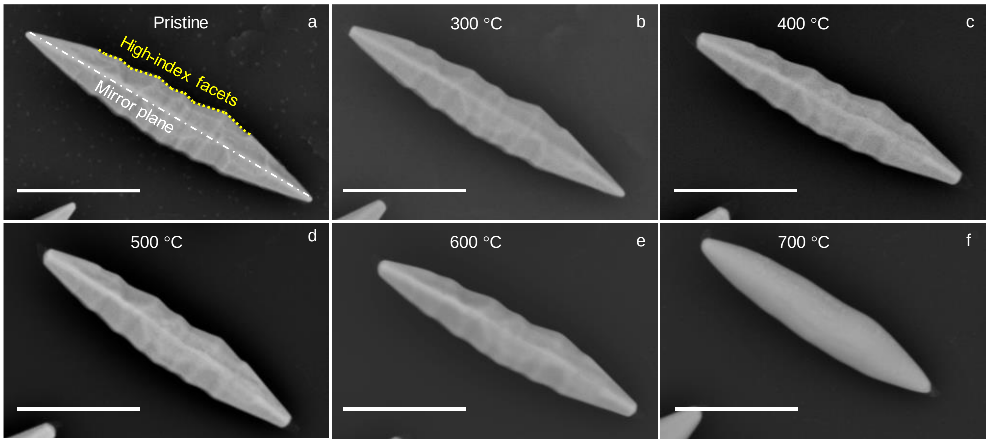

Figure S7. Ex-situ temperature dependent SEM images of a crystallite. (a) Pristine crystallite along with the marked high-index facets and the mirror plane. A collection of crystallites was annealed for 20 min at each temperature and later, imaged under SEM (a-f). Scale bar, 1 µm. The same crystallite in (a) is shown for better comparison.

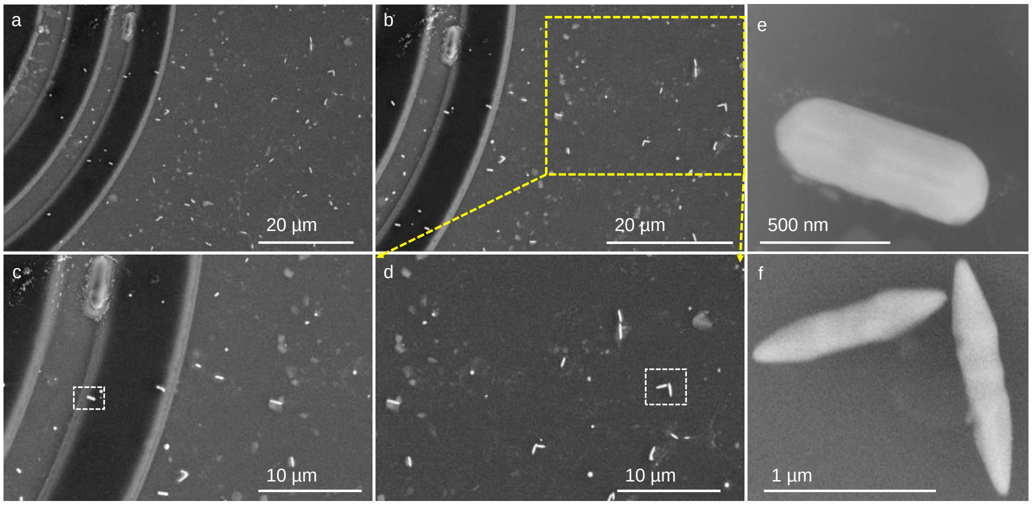

Figure S8. (a-d) SEM images of the heating coil and the crystallites inside the coil at different magnifications are shown. The magnified views of the crystallites marked in (c,d) are shown in (e,f), respectively. SEM images of Au crystallites (e) inside the heating coil and (f) ~ 35 µm away from the edge of the coil, exhibiting two different morphologies. The heating appears to be local, since the morphologies of the crystallites are nearly intact in (f), indicating dropping of the temperature.

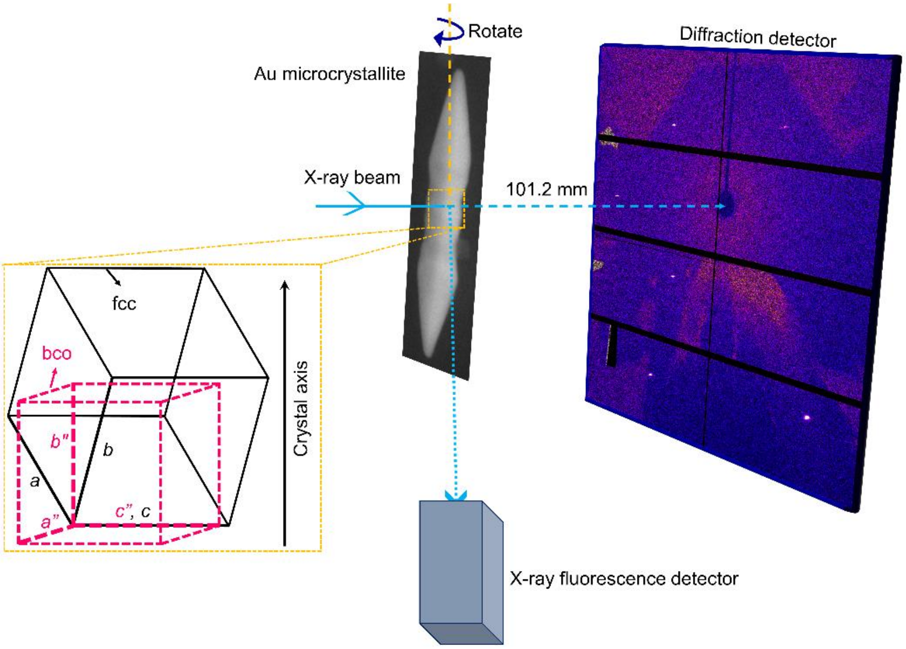

Figure S9. Schematic representation of the diffraction geometry. The orientation of face-centered cubic (fcc) and body-centered orthorhombic (bco) unit cells in the crystallite with (*a*, *b*, *c*) and (*a''*, *b''*, *c''*) are their corresponding axes, respectively.

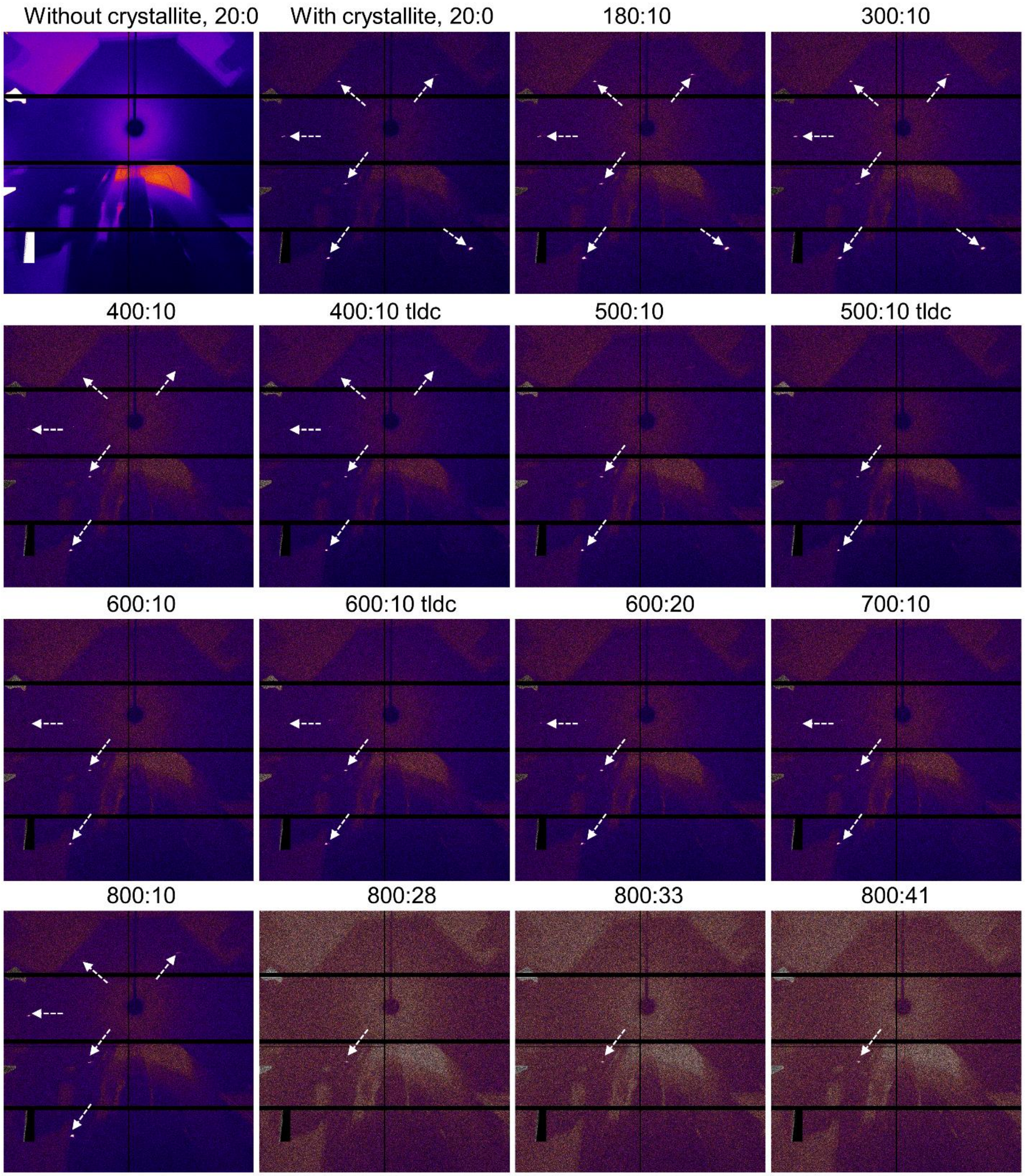

Figure S10. Integrated diffraction data as collected for the thermally treated crystallite using an Eiger X 4M detector at 20 °C and 800 °C. The diffraction spots have been indicated by arrows. The annealing and data collection details are written on top of each panels. Diffraction data collected without crystallite in the X-ray beam is referred to as the background image. Here, before

integration, all the diffraction data has been background subtracted. All the images are on the same color scale, except the without crystallite which is on 200 times lower scale.

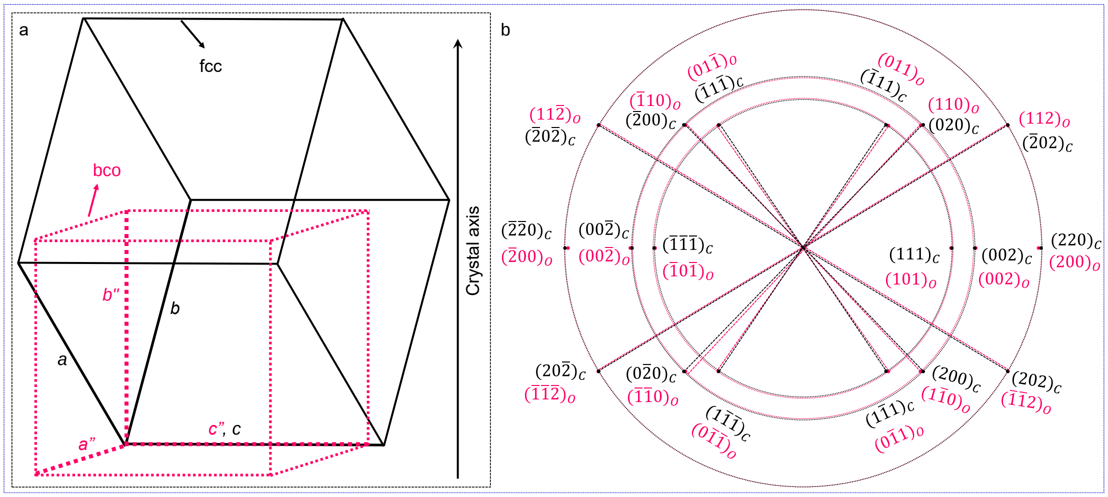

Figure S11. Visualizing the unit cells and simulating the corresponding reflections. (a) The orientation of face-centered cubic (fcc) and body-centered orthorhombic (bco) unit cells in the crystallite with (*a*, *b*, *c*) and (*a"*, *b"*, *c"*) are their corresponding axes, respectively. The *b"* axis is parallel to the goniometer y-axis and also to the crystal growth direction. The *c* and *c"* axes overlap and lie parallel to the horizontal plane. Rotation between both unit cells is 45° (along the *c*-axes), where *c"* > *a"* and *b"*. The schematic is not adjusted for scale. (b) Simulated and overlaid diffraction patterns from both fcc and bco unit cells with 360° rotation around ϕ.

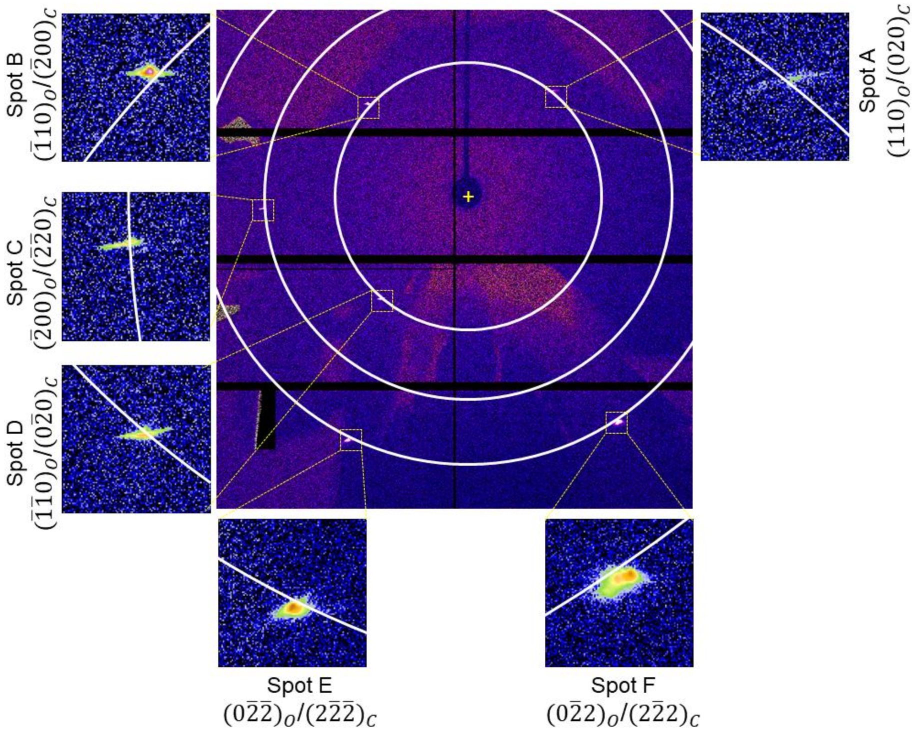

Figure S12. fcc circles with q of (002), (220) and (222) are drawn over the diffraction spots collected from pristine crystallite. The magnified views show the arc of the circle passing through the spot. Background subtracted detector image and the corresponding magnified views of the diffraction spots are shown.

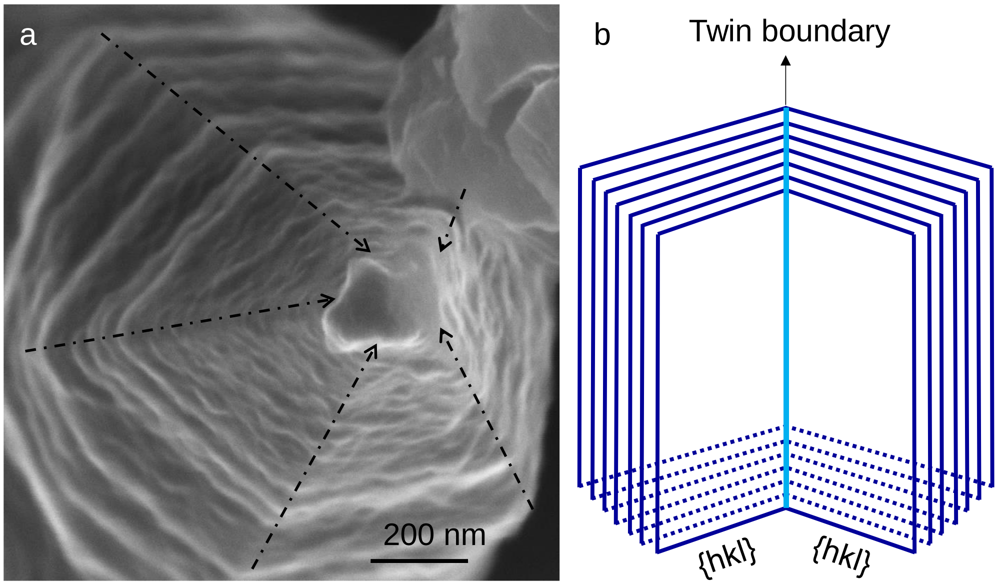

Figure S13. (a) Penta-twinned tip of the crystal. The five twin boundaries are shown by black dash-dotted arrow. (b) Schematic representation of twin boundary. The twin boundary acts as mirror plane and thereby enabling to satisfy mirror reflections.

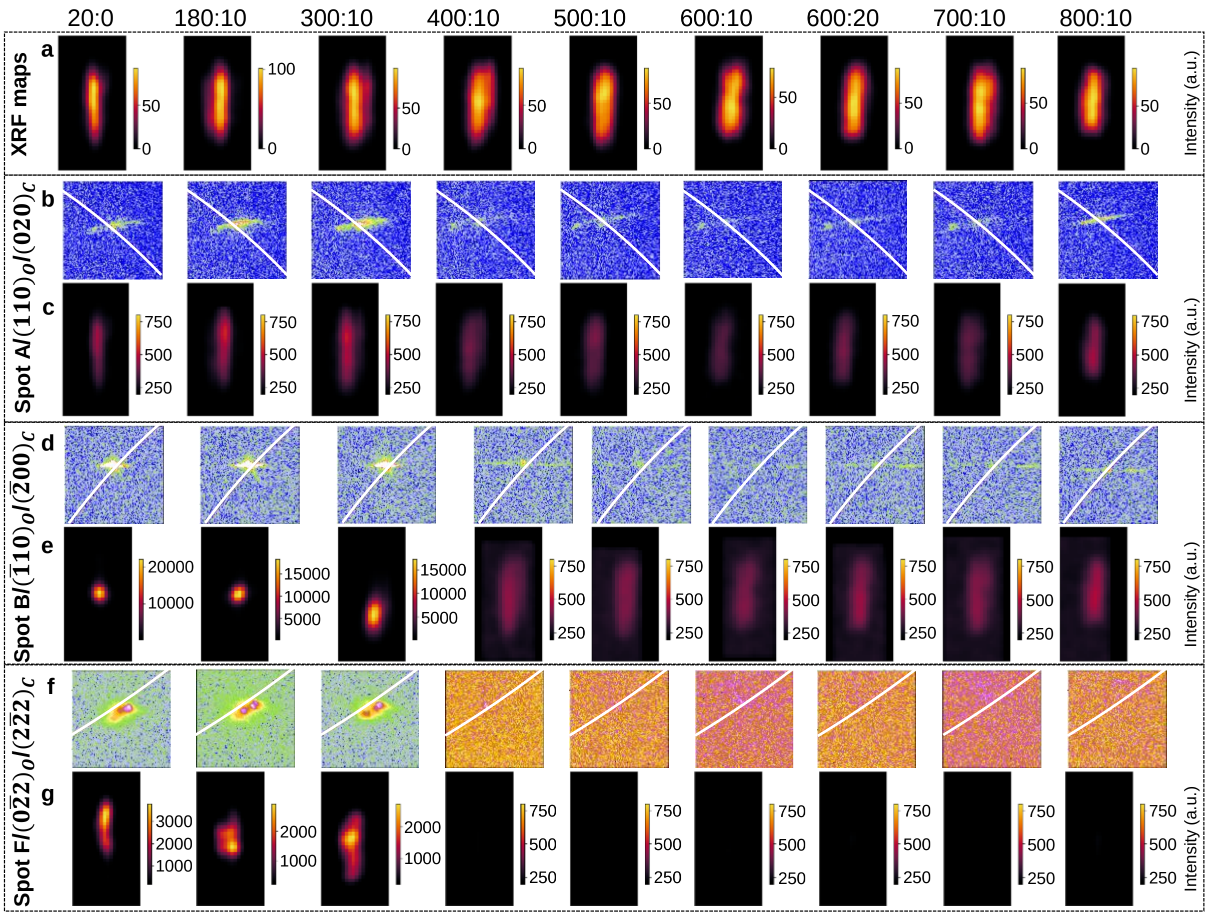

Figure S14. Temperature dependent (a) XRF maps, (b,d,f) diffraction spots A, B and F and (c,e,g) the corresponding diffraction maps, respectively. Pixel windows (100 × 100) in (b,d,f). The white arcs of the circles, corresponding to the *q* value of fcc(002) and fcc(222), pass through the spots. The *q* values were derived from the standard fcc Au lattice parameters (3.0804 $Å^{-1}$ and 5.3355 $Å^{-1}$). The annealing and data collection details are written on top of the panel.

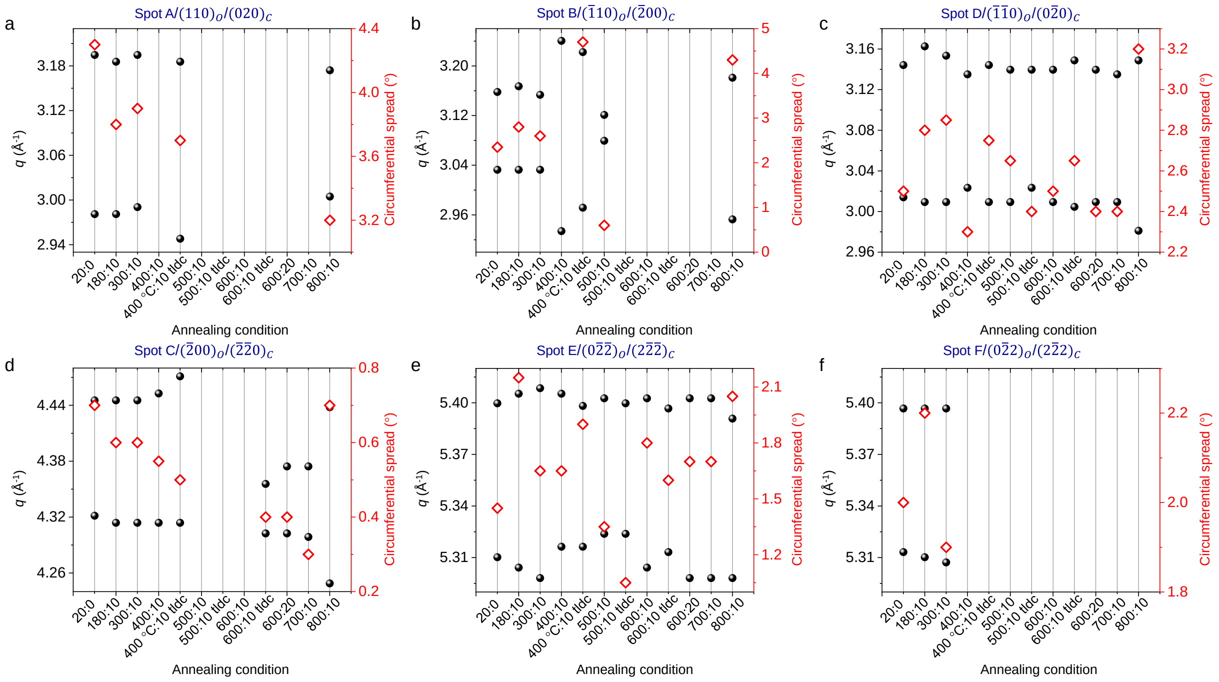

Figure S15. Calculating the spread of the various diffraction spots at different annealing conditions. The diffraction spots, at both pre-annealed and post-annealed conditions (see Figures 4 and S13) display highly asymmetric intensity spreads both circumferentially and radially. Here, for each spot, the q_{max} and q_{min} values extracted after examining the intensity spread at each annealing temperature. Similarly, the intensity spread observed circumferentially was also measured for each spot. The spreads (difference between the q_{max} and q_{min}) along the radial direction are much higher than ±0.0045 Å^{-1}, the instrumental resolution. Again, the circumferential spreads are substantially higher which can arise due to the co-presence of oriented domains with different lattice parameters or misoriented domains with similar lattice parameters. Spots with significant intensities are shown here.

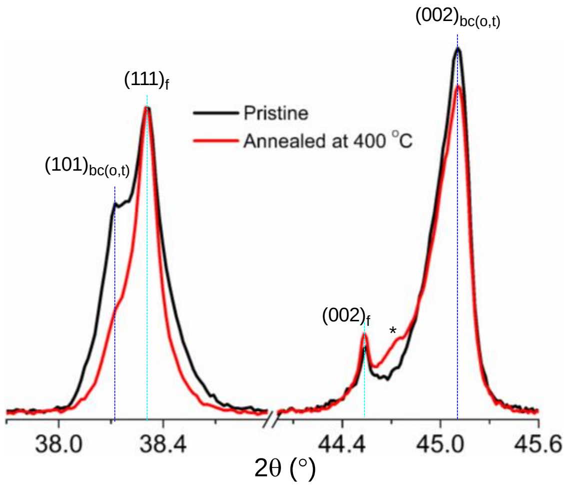

Figure S16. Laboratory XRD pattern of body-centered orthorhombic and tetragonal lattices (together called as bc(o,t)) rich Au crystallites annealed at 400 °C for 30 min along with the pristine pattern. * denotes the additional peak formed during thermal annealing, which was absent in the pristine pattern. f represents fcc.

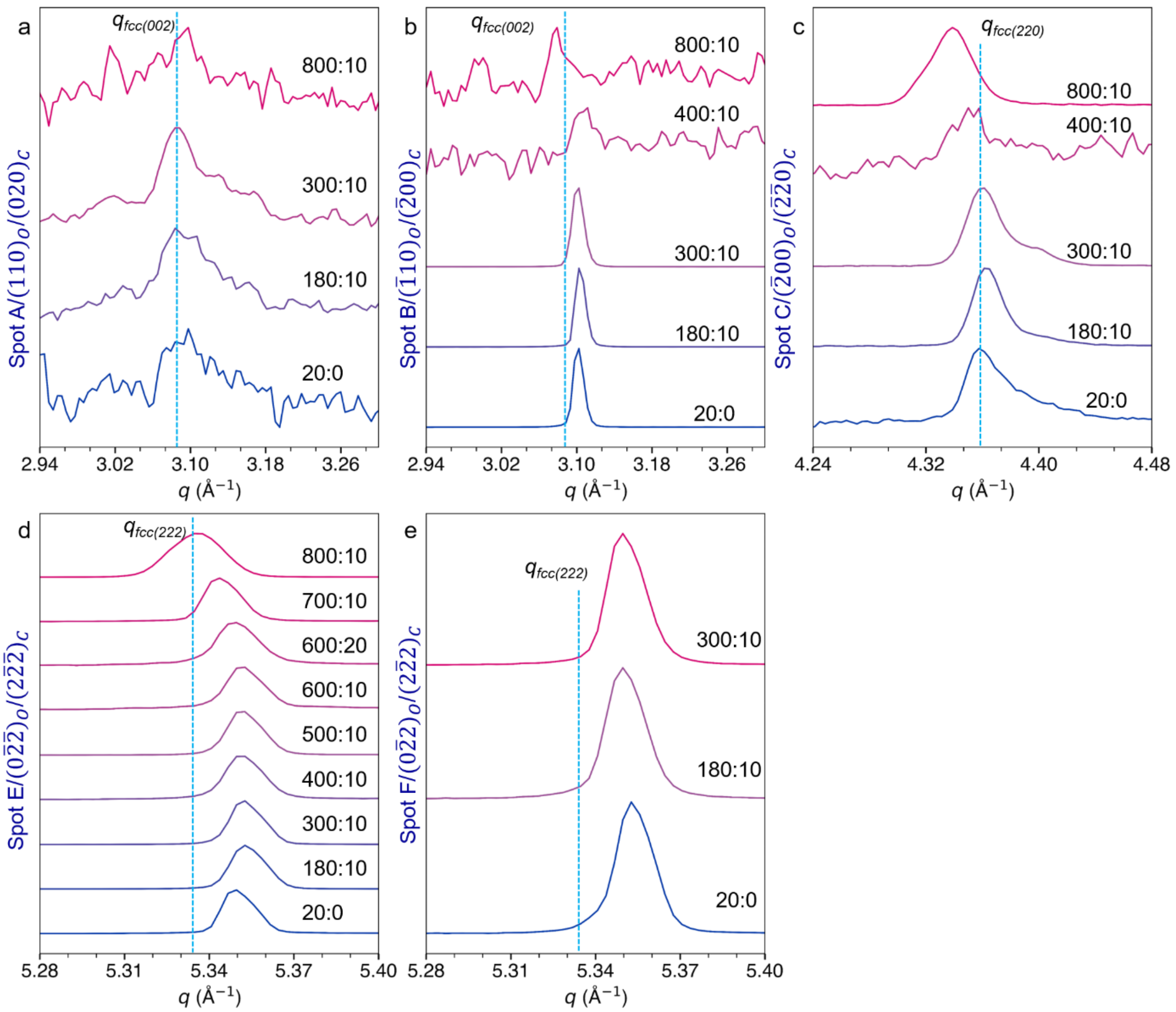

Figure S17. (a-e) Line profiles of the diffraction spots (integrated along both y and z) A, B, C, E and F, respectively drawn along the radial direction for various temperatures. The intensities have been normalized. Post 800 °C treatment (800:10), peak positions shifted to the lower q values. Line profiles with significant intensities are shown here.

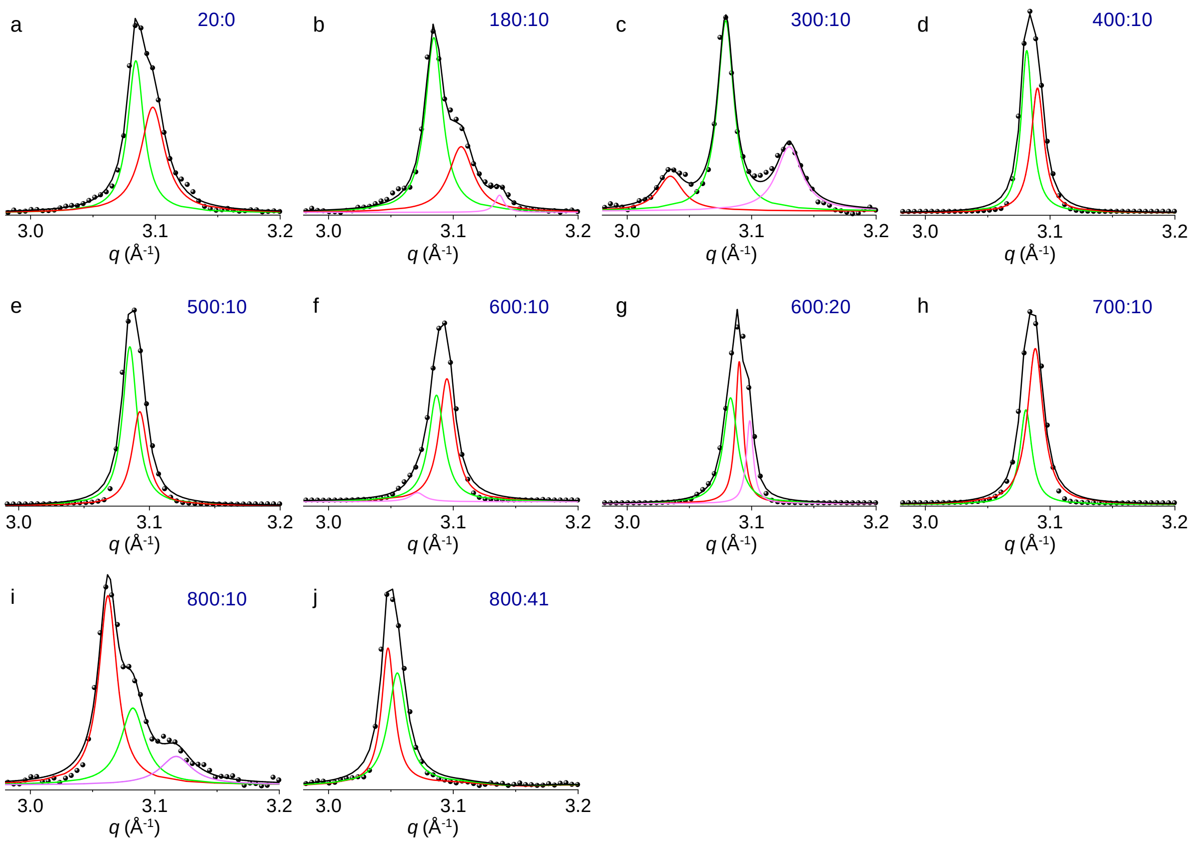

Figure S18. (a-j) Peak fitting of Spot $\mathrm{D}/(\bar{1}\bar{1}0)_O/(0\bar{2}0)_C$ at different annealing conditions. Black spheres represent experimental data. The fitted peaks are in green, red and magenta and their sum is represented in black solid curve. Green colored curve represents fcc contribution. The annealing and data collection details are written alongside the plots. Areas under the curves are considered for the bco fraction calculation. Therefore, areas under the red and magenta colored curves contribute towards bco calculation while area under green colored curve for fcc.

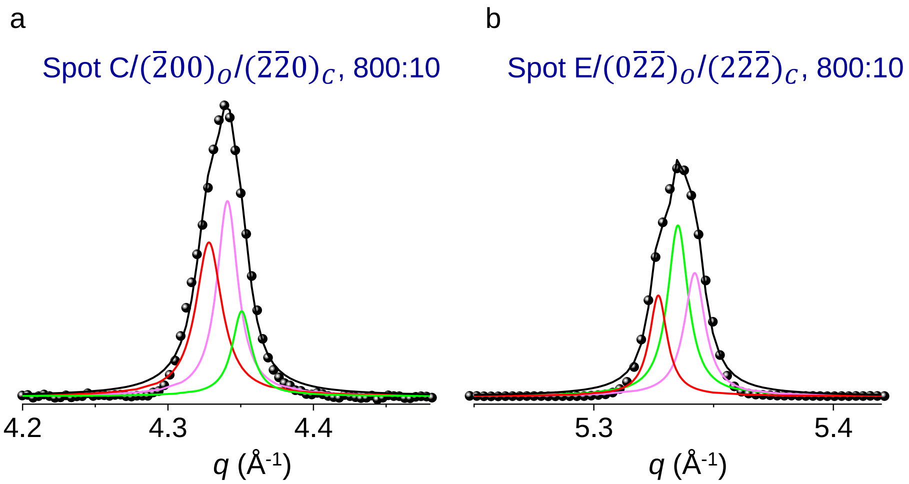

Figure S19. Peak fitting of spot C and E after 800 °C. Black spheres curve represents experimental data. The fitted peaks are in green, red and magenta and their sum is represented in black solid curve. Green colored curve represents fcc contribution. The annealing temperature and time details are written alongside the plots. Areas under the curves are considered for the bco fraction calculation. Therefore, areas under the red and magenta colored curves contribute towards bco calculation while area under green colored curve for fcc.

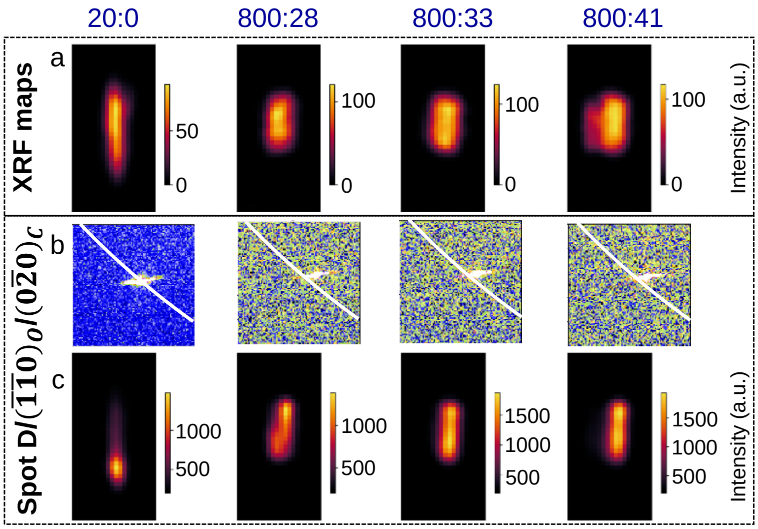

Figure S20. Concurrent heating and data collection. Temperature-dependent (a) XRF maps, (b) diffraction spot D and (c) the corresponding diffraction maps, respectively. Pixel windows (100 × 100) in (b). The white arc of the circle, corresponding to the q value of fcc(002), passes through the spots. The annealing and data collection details are written on top of the panel. Here, data was collected at 800 °C while annealing was continuing at the same temperature.

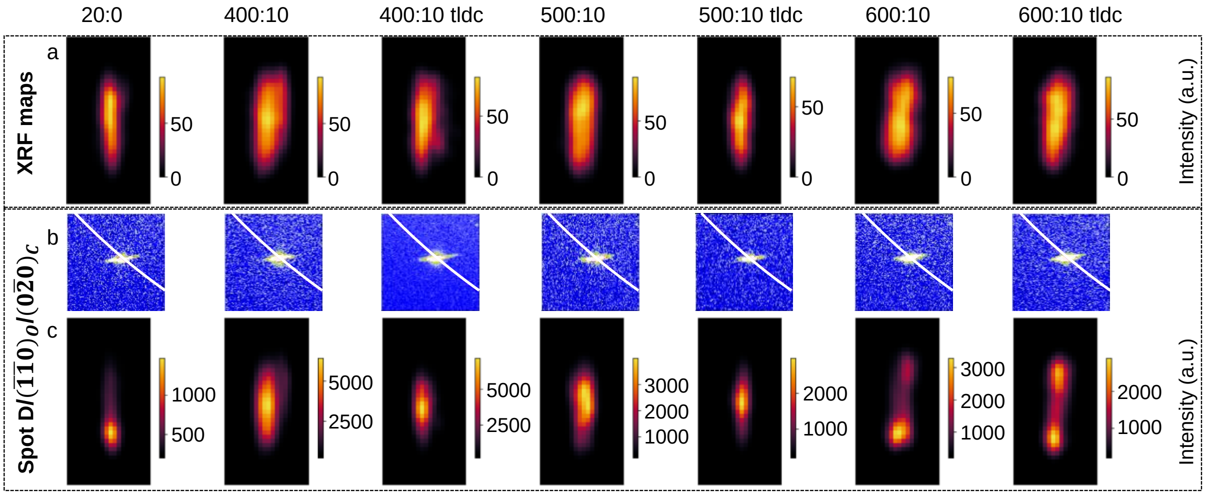

Figure S21. Temperature dependent (a) XRF maps, (b) diffraction spot D and (c) the corresponding diffraction maps, respectively. Pixel windows (100 × 100) in (b). The white arc of the circle, corresponding to the *q* value of fcc(002), passes through the spots in (b). The annealing and data collection details are written on top of the panel. Annealing allows enhancement in diffraction area. However, the time lapse data (tldc) show reduction in diffraction area compared to the data collected immediately after annealing. Therefore, this shows that the crystal at this temperature is not yet in equilibrium.

Table S1. Estimating q values. q values were quantified based on the centroids of the line profiles drawn over the diffraction spots, where the detector resolution is ± 0.0045 $Å^{-1}$. The zoomed-in views of the diffraction spots with (100 × 100) pixels exhibiting the wide spread along circumferential and radial directions.

Label	Diffraction spot	Line profile, intensity (a.u.) vs. q ($Å^{-1}$)	q ($Å^{-1}$)
A	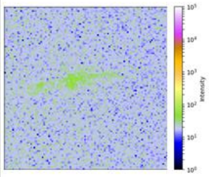	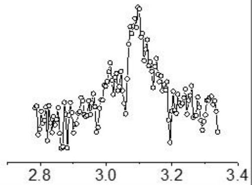	3.0962
B	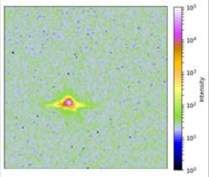	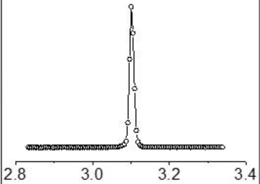	3.1008
C	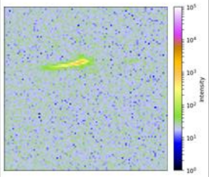	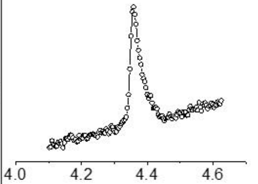	4.3578
D	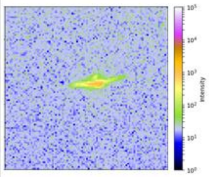	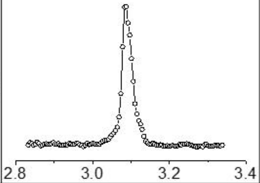	3.0877
E	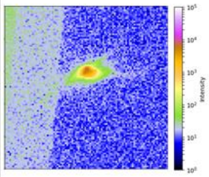	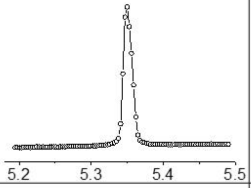	5.3496
F	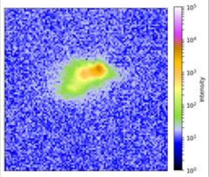	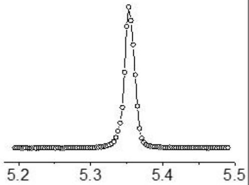	5.3526

Table S2. q spread of pristine crystallite at 20 °C.

Spot	q_{min} (Å^{-1})	q_{max} (Å^{-1})	*Azimuthal spread (°)*	q_{mean} (Å^{-1})	Lowest q_{min} (Å^{-1})	Highest q_{max} (Å^{-1})
A	2.9811	3.1947	4.3	3.0874	2.9811	3.1947
B	3.0327	3.1580	2.35			
D	3.0140	3.1442	2.5			
C	4.3215	4.4453	0.7	4.3834	4.3215	4.4453
E	5.3102	5.3997	1.45	5.3549	5.3102	5.3997
F	5.3132	5.3967	2			

Table S3. {*hkl*} and q values from our previous reports for fcc, bct and bco lattices, respectively. The lattice parameters of fcc: $a = b = c = 4.0810$ Å; bct: $a = b = 2.9069$ Å, $c = 4.0430$ Å; bco: $a = 2.9178$ Å, $b = 2.8930$ Å, $c = 4.0314$ Å, respectively.

fcc		*bct*		*bco*	
**{*hkl*}**	***q*** **(Å^{-1})**	**({*hkl*}**	***q*** **(Å^{-1})**	**{*hkl*}**	***q*** **(Å^{-1})**
(111)	2.6677	(101)	2.6632	(101)	2.6593
(002)	3.0804	(110)	3.0580	(011)	2.6742
(220)	4.3564	(002)	3.1094	(110)	3.0596
(113)	5.1083	(200)	4.3246	(002)	3.1183
(222)	5.3355	(112)	4.3611	(200)	4.3085
		(202)	5.3264	(020)	4.3454
				(112)	4.3687
				(202)	5.3186
				(022)	5.3485

Table S4. Comparison of obtained *q* values with different lattices.

**Spot**	$\mathbf{q_{mean}}$ **(Å^{-1})**	**Experimental error involved (Å^{-1})**	**Possible *(hkl)***	**Previously reported *q* (Å^{-1})**	**Angle between the extended reflections (°)**			**Remarks**
					**Spots under consideration**	**min**	**max**	
A, B, D	3.0874	-0.0294<br>**-0.0278**<br>**-0.0070**<br>0.0220<br>0.0309	*bct*{110}<br>***bco*{110}**<br>***fcc*{002}**<br>*bct*{002}<br>*bco*{002}	3.0580<br>**3.0596**<br>**3.0804**<br>3.1094<br>3.1183	A,B<br>B,D<br>D,A	83.2<br>90.35<br>175.9	89.85<br>95.2<br>182.7	Four reflections would be generated from *fcc*{002} and *bct*{110} with an inter-reflection angle – 90°. Only reflections from *bco*{110} could accommodate reflections with an angular spread – deviated from perfect 90°.<br>Angle between mirror reflections will be 180° and the deviation accounts for the presence of multiple domains.<br>For simplicity, only *fcc* and *bco* are considered.
C	4.3834	**-0.0749**<br>-0.0588<br>-0.0380<br>**-0.0270**<br>-0.0223<br>-0.0147	***bco*{200}**<br>*bct*{200}<br>*bco*{020}<br>***fcc*{220}**<br>*bct*{112}<br>*bco*{112}	**4.3085**<br>4.3246<br>4.3454<br>**4.3564**<br>4.3611<br>4.3687	---	---	---	With the orientation of the unit cells, *fcc*{220} and *bco*{200} are the possibilities.
E, F	5.3549	-0.0363<br>-0.0285<br>**-0.0194**<br>**-0.0064**	*bco*{202}<br>*bct*{202}<br>***fcc*{222}**<br>***bco*{022}**	5.3186<br>5.3264<br>**5.3355**<br>**5.3485**	E,F	58.1	61.55	With the orientation of the unit cells, *fcc*{222} and *bco*{022} are the possibilities.

Table S5. Calculated lattice parameters of bco crystallite. From the q spread, q_{min}, q_{max} and q_{mean} (for the symmetry reflections) were obtained. Using the q values (in bold) in Table S4, lattice parameters were calculated.

Temperature	d	$d_{\{110\}O}$ (Å)			**Mean $d_{\{110\}O}$ (Å)**	$d_{\{200\}O}$ (Å)	$d_{\{022\}O}$ (Å)		**Mean $d_{\{022\}O}$ (Å)**	a_{bco} (Å)	b_{bco} (Å)	c_{bco} (Å)
		Spot A	Spot B	Spot D		Spot C	Spot E	Spot F				
20 °C	d_{min}	2.1085	2.0726	2.0855	**2.0888**	**1.4545**	1.1837	1.1830	**1.1833**	2.9090	3.0011	3.8483
	d_{max}	1.9675	1.9904	1.9991	**1.9856**	**1.4140**	1.1640	1.1647	**1.1643**	2.8280	2.7885	4.2327
800 °C	d_{min}	2.0920	2.1287	2.1085	**2.1097**	**1.4794**	1.1864	---	**1.1864**	2.9588	3.0089	3.8587
	d_{max}	1.9803	1.9760	1.9962	**1.9841**	**1.4163**	1.1660	---	**1.1660**	2.8326	2.7800	4.2838

References:

(1) Mettela, G.; Sorb, Y. A.; Shukla, A.; Bellin, C.; Svitlyk, V.; Mezouar, M.; Narayana, C.; Kulkarni, G. U. Extraordinarily Stable Noncubic Structures of Au: A High-Pressure and -Temperature Study. *Chem. Mater.* **2017**, *29*, 1485–1489.
